# Macrophages Modulate Migration and Invasion of Human Tongue Squamous Cell Carcinoma

**DOI:** 10.1371/journal.pone.0120895

**Published:** 2015-03-26

**Authors:** Emma Pirilä, Otto Väyrynen, Elias Sundquist, Kaisa Päkkilä, Pia Nyberg, Sini Nurmenniemi, Virve Pääkkönen, Paula Pesonen, Dan Dayan, Marilena Vered, Lars Uhlin-Hansen, Tuula Salo

**Affiliations:** 1 Oulu Center for Cell-Matrix Research, University of Oulu, Oulu, Finland; 2 Department of Diagnostics and Oral Medicine, Institute of Dentistry, University of Oulu, Oulu, Finland; 3 Oulu Medical Research Center, University of Oulu and Oulu University Hospital, Oulu, Finland; 4 Department of Community Dentistry, Institute of Dentistry, University of Oulu, Oulu, Finland; 5 Department of Oral Pathology and Oral Medicine, School of Dental Medicine, Tel Aviv University, Tel Aviv, Israel; 6 Institute of Pathology, The Chaim Sheba Medical Center, Tel Hashomer, Tel Aviv, Israel; 7 Institute of Medical Biology, Faculty of Health Sciences, University of Tromsø, Tromsø, Norway; 8 Department of Pathology, University Hospital of Northern Norway, Tromsø, Tromsø, Norway; 9 Oulu University Hospital, Oulu, Finland; 10 Department of Pedodontics, Cariology and Endodontology, University of Oulu, Oulu, Finland; The University of Hong Kong, CHINA

## Abstract

Oral tongue squamous cell carcinoma (OTSCC) has a high mortality rate and the incidence is rising worldwide. Despite advances in treatment, the disease lacks specific prognostic markers and treatment modality. The spreading of OTSCC is dependent on the tumor microenvironment and involves tumor-associated macrophages (TAMs). Although the presence of TAMs is associated with poor prognosis in OTSCC, the specific mechanisms underlying this are still unknown. The aim here was to investigate the effect of macrophages (Mfs) on HSC-3 tongue carcinoma cells and NF-kappaB activity. We polarized THP-1 cells to M1 (inflammatory), M2 (TAM-like) and R848 (imidazoquinoline-treated) type Mfs. We then investigated the effect of Mfs on HSC-3 cell migration and NF-kappaB activity, cytokine production and invasion using several different in vitro migration models, a human 3D tissue invasion model, antibody arrays, confocal microscopy, immunohistochemistry and a mouse invasion model. We found that in co-culture studies all types of Mfs fused with HSC-3 cells, a process which was partially due to efferocytosis. HSC-3 cells induced expression of epidermal growth factor and transforming growth factor-beta in co-cultures with M2 Mfs. Direct cell-cell contact between M2 Mfs and HSC-3 cells induced migration and invasion of HSC-3 cells while M1 Mfs reduced HSC-3 cell invasion. M2 Mfs had an excess of NF-kappaB p50 subunit and a lack of p65 subunits both in the presence and absence of HSC-3 cells, indicating dysregulation and pro-tumorigenic NF-kappaB activation. TAM-like cells were abundantly present in close vicinity to carcinoma cells in OTSCC patient samples. We conclude that M2 Mfs/TAMs have an important role in OTSCC regulating adhesion, migration, invasion and cytokine production of carcinoma cells favouring tumor growth. These results demonstrate that OTSCC patients could benefit from therapies targeting TAMs, polarizing TAM-like M2 Mfs to inflammatory macrophages and modulating NF-kappaB activity.

## Introduction

Oral squamous cell carcinoma (OSCC) is the eight most common cancer in the world. Annually, 640 000 new oral cancers are reported worldwide [1]. The World Health Organization expects a worldwide increase in incidence in the next few decades. The most common site affected by OSCC is the tongue and at this site OSCC is particularly aggressive. The 5-year survival rate for tongue cancer has remained at approximately 50% without significant improvements [2]. An alarming report showed that tongue cancer is increasing especially in young adults and none of the typical etiological factors such as tobacco, alcohol or human papilloma virus can be accounted for [3].

Currently, there is no specific marker in clinicopathological use to identify aggressive early stage OSCC tumors [4]. In recent years, it has been accepted that to effectively treat cancer, the tumor must be considered as an entity containing both the cancer cells and the surrounding tissue which together forms the tumor microenvironment (TME). In certain types of cancer, including squamous cell carcinomas, the TME may even be dominant over cancer cell malignancy [5]. TME contains a mixture of heterotypic cells such as cancer-associated fibroblasts (CAFs), smooth muscle cells, endothelial cells, neutrophils, lymphocytes and macrophages [6]. Of these, especially CAFs and macrophages (Mfs) are known to promote tumor progression in tongue cancer [7–9].

Links between cancer and inflammation were made already in the 19th century by Rudolf Virchow, demonstrating that tumor tissues includes chronic inflammation and contain leukocytes [6]. The inflammatory infiltrate of a TME is an essential source of cytokines, chemokines, growth factors, angiogenetic factors and enzymes produced by inflammatory cells associated with tumor growth and progression. This type of inflammatory infiltrate compromise a “bad inflammation” in contrast to components of the inflammatory infiltrate that represent an anti-tumorigenic force and a “good inflammation” [8]. This opens up new prognostic and therapeutic possibilities because the tumor-associated stromal cells are genetically more stable than cancer cells and should therefore also be less prone to develop chemoresistance. However, in some instances the tumor stromal cells can also contribute to tumor chemoresistance, so targeting stromal cells or their products may be a viable strategy [10,11].

Macrophages are a heterogenous population of innate myeloid cells derived from monocytic precursors in the blood and undergo specific differentiation depending on the signaling in the tissue. Mfs are highly plastic in regard to phenotypes but can roughly be divided into two subtypes based on surface receptors, cytokine production and reactivity: the classically activated inflammatory M1 Mfs and the alternatively activated M2 Mfs. Cancer-associated inflammation includes the so called tumor-associated macrophages (TAMs) which are generally thought to resemble M2-type Mfs. TAMs suppress the Th1 immune response, possess anti-cytotoxic effects, promote angiogenesis and thus benefit survival and spreading of tumor cells [12,13]. TAMs release reactive oxygen species, tumor necrosis factor (TNF)-α, Interleukin [IL]-6 and IL-1β, promoting DNA damage, transformation and cancer cell survival. The abundance of TAMs in TME is associated with poor prognosis in ovarian, breast, bladder, prostate and renal cell cancer but not in colon and gastric cancer [14]. The presence of TAMs are associated with a poor prognosis in oropharyngeal and oral cavity cancers [8,15–18]. A higher percentage of TAMs in metastatic OSCC in contrast to non-metastatic OSCC has been found and also contributed to decreased patient survival [18]. The TME and inflammation in tongue carcinoma is not very well characterized or studied but is of critical importance for metastasis [19].

The transcription factor nuclear factor kappa B (NF-κB) is an important and critical regulator of inflammation. NF-κB is a dimer formed from multiple subunits consisting of p65 (Rel A), Rel B, c-Rel, p105/p50 (NF-κB1), and p100/p52 (NF-κB2). In the classical NF-κB signaling pathway, the p50/p65 subunits are stored in the cytosol bound to their inhibitor IκBα. Upon activation, IκB kinase 2 phosphorylates IκBα which leads to ubiquitination and degradation of the inhibitor complex. p50/p65 then translocates into the nucleus, binds to consensus DNA sequences and activates transcription of target genes [20]. Increased oral carcinoma cell migration and invasion was associated with enhanced NF-κB activity and inhibition suppressed both invasion and proliferation of OSCC cells [21]. Etiological factors activating oral carcinogenesis such as tobacco and alcohol are known to activate NF-κB signaling. However, recent research demonstrate that while inflammation and subsequent NF-κB activation may contribute to carcinogenesis, the TME becomes tolerated which favors tumor progression [22]. In particular, the tolerization of recruited TAMs induces the formation of NF-κB p50 homodimers in the nucleus which represses NF-κB signaling and leads to down-regulation of NF-κB induced genes and upregulation of so called non-tolerable genes which are thought to contribute to tumor progression [23].

Imiquimod is an imidazoquinoline which induces immune responses and is used for superficial treatment of basal and squamous cell carcinomas of the skin. Imiquimod is a toll-like-receptor (TLR)-7/8 agonist activating the myeloid differentiation primary response protein 88 (MyD88) and subsequently leading to NF-κB activation [24]. Imiquimod is known to act on skin carcinomas through the modulation of the immune cells and polarization of Mfs in the TME [24,25]. Recently, imiquimod-containing mucoadhesive films have been developed for the treatment of OSCC [26].

Here we studied the interaction of aggressive HSC-3 tongue carcinoma cells and THP-1 Mfs polarized to type M1 and M2 Mfs. In addition, we wanted to study the direct effect of imiquimod on macrophage polarization *in vitro*. We found that in monolayer co-cultures, Mfs changed the migratory properties of HSC-3 cells and in addition, both cell types exhibited altered cell surface markers, cytokine content and NF-κB activity. By using the human hypoxic 3D invasion model developed by our group [27,28] we also found that M2 Mfs, but not M1 Mfs, induced invasion of HSC-3 cells. The imiquimod-polarized Mfs did not affect HSC-3 cell invasion, however, they reduced proliferation of HSC-3 cells.

## Materials and Methods

### Cell culture

HSC-3 tongue carcinoma cells (Japanese Collection of Research Bioresources Cell Bank. Cat.no: JCRB0623) were cultured in DMEM/F12 supplied with 10% fetal bovine serum (both from Life Technologies, CA, USA), 50 μg/ml ascorbic acid, 0.4 μg/ml hydrocortisone, 100 U/ml penicillin, 100 μg/ml streptomycin and 250 ng/ml Fungizone (all from Sigma-Aldrich Co.LLC, MO, USA).

THP-1 leukemia cells (ATCC, VA, USA. Cat.no. TIB-202) were cultured in advanced RPMI supplied with 10% fetal bovine serum (both from Life Technologies, CA, USA) supplemented with 2 mM L-Glutamine (Life Technologies, CA, USA), 100 U/ml penicillin, 100 μg/ml streptomycin and 250 ng/ml fungizone (all from Sigma-Aldrich Co. LLC, MO, USA). For both cell lines, passages only up to 30 were used. Both cell lines have been characterized by the manufacturers. In addition, they were subjected to short tandem repeat-profiling by Identicell (Århus, Denmark) and found to match HSC-3 and THP-1 cell lines (S1 and S2 Tables respectively).

THP-1 cells were polarized into Mfs as previously described [54] by priming for 6 h with 200 ng/ml phorbol 12-myristate 13-acetate (Sigma-Aldrich Co.LLC, MO, USA). Cells were then polarized toward M1-type by incubating with 20 ng/ml interferon-γ (ProSpec, Ness-Ziona, Israel) and 100 ng/ml lipopolysaccharide (Sigma-Aldrich Co.LLC, MO, USA) for another 18 hours or toward M2-type by incubating with 20 ng/ml Il-4 and 20 ng/ml IL-13 (both from ProSpec, Ness-Ziona, Israel) for 18 hours. In addition, we tested the effect of an imidazoquinoline-compound R848, which act as a TLR7/TLR8-agonist for the polarization of THP-1 cells to Mfs. THP-1 cells were first primed with PMA as described above and then incubated with 5 ng/ml R848 (InvivoGen, CA USA) for another 18 hours.

For co-culture experiments, HSC-3 cells were labeled with Vybrant CM-Dil (red) and Mfs were labeled green with Vybrant DiO (both stains from Life Technologies, CA, USA). HSC-3 cells and polarized Mfs were seeded to TC Lab-Tek Chamber slides (Thermo Fisher Scientific, MA, USA) at approximately a 1:2 ratio of HSC-3 cells and Mfs. Incubation was done in 50% HSC-3 medium/50% THP-1 medium w/wo 10% FBS. For some experiments, conditioned media (CM) from HSC-3 cells, polarized Mfs or co-cultures were collected. Cells were washed with phosphate-buffered saline (PBS) and then serum free (SF)-medium supplemented with 0.1% bovine serum albumin (from Sigma Aldrich Co.LLC, MO, USA) was added to the cells. Cells were incubated o/n where after media was collected. In some experiments, cells were supplied with normal growth medium and medium collected at additional days, accordingly.

### Efferocytosis

2500 Vybrant CM-Dil-labeled HSC-3 cells and 5000 Vybrant DiO-labeled Mfs were co-cultured in TC Lab-Tek Chamber slides (Thermo Fisher Scientific, MA, USA). The cells were allowed to attach o/n in normal growth medium. Thereafter cells were washed with PBS, and medium changed to SF Optimem (Life Technologies, CA, USA). The following experimental groups were created (n = 4): co-cultures of HSC-3 cells and Mfs (M1 Mf, M2 Mf, R848 Mf) were incubated with either DMSO (1:20 000), 0.3 mM Amiloride (Sigma-Aldrich Co.LLC, MO, USA), 3 mM Amiloride, 5 μM NF-κB inhibitor BAY 11-7082 (MerckMillipore, MA, USA) or 3 mM Amiloride plus 5 μM BAY 11-7082. Also monocultures of HSC-3 cells and Mfs were incubated with the same experimental molecules. Incubation was continued for up to 7 days and monitored once every day with the Evos FL Cell Imaging System (Life Technologies, CA, USA).

### Antibody array

Serum-free media from the mono and co-culture experiments (day 2) were collected as described above. The media were analyzed with RayBio Human Cytokine Antibody Array G5 (RayBiotech, GA, USA) for the detection of 80 cytokines. The values were normalized and background was reduced. Thereafter the values were normalized against sample protein amount.

### Horizontal migration analyses

Horizontal cell migration was first tested with the Oris Pro cell migration assay (Platypus technologies, WI, USA). Vybrant CM-Dil (red) HSC-3 and Vybrant DiO (green) THP-1 cells (5000 each) were seeded onto Oris Pro cell migration 96-well plates and allowed to attach with inserts overnight. Thereafter inserts were removed, the cells washed with PBS and medium with 1% serum was added. Migration was monitored for 48 hours by photographing wells with an Evos FL Cell Imaging System (Life Technologies, CA, USA) and the area of migrated, labeled cells was analyzed with QWin3 Software (Leica Microsystems, Wetzlar, Germany). Each experimental group was done in quadruplicate.

In another experiment, horizontal cell migration was tested with Ibidi inserts (Ibidi, Planegg/Martinsried, Germany). 20 000 cells were applied and allowed to grown to confluence with the inserts forming an empty area in the middle of the plate (similar setting to the Oris Pro cell migration 96-well plates). Inserts were removed and cell migration into the empty area was monitored using an Evos FL Cell Imaging System (Life Technologies, CA, USA). The empty area without cells was measured with the ImageJ software (Research Services Branch, National Institute of Mental Health, Maryland, USA). Each experimental group was done in triplicate. The effect of M1 and M2 conditioned medium was tested, in addition to the effect of NF-κB inhibitor BAY 11-7082 at 10 μM.

### Adhesion assay

6000 HSC-3 cells were seeded to a 96-well plate coated with fibronectin (10 μg/ml in PBS, Sigma Aldrich Co.LLC, MO, USA). Cells were seeded in SF medium supplemented with either 10% FBS, 0.1% BSA, DMSO (1:20 000), 5 μM BAY 11-7082, M1 Mf- CM, M2 Mf-CM or R848 Mf CM (n = 6 per group). Cells were allowed to attach for 2 hours where after the wells were washed with PBS and the cells were fixed with 10% trichloracid (TCA, from Sigma Aldrich Co.LLC, MO, USA), washed with water and air dried. The wells were then stained with 0.1% crystal violet (Sigma Aldrich Co.LLC, MO, USA). The cell density was determined by measuring the absorbance at 540 nm with a Victor X3 plate reader (Perkin Elmer, CA, USA).

### Chemotactic migration assays

For chemotactic migration assays, Transwell membrane inserts (Corning Inc., MA, USA) were coated with 10 μg/ml fibronectin (in PBS and from Sigma Aldrich Co.LLC, MO, USA) for 2 hours at 37°C, blocked for 2 hours at room temperature with BSA/PBS and thereafter washed with PBS. 25 000 HSC-3 cells were seeded to the upper chambers and 250 000 HSC-3-cells, M1- or M2- or PMA-Mfs were seeded to the bottom of the lower chamber. In addition some lower chambers were left without cells.

In a second Transwell experiment 50 000 M1-, M2- or PMA Mfs or HSC-3 cells were seeded to the upper chambers and 240 000 HSC-3 cells or THP-1 cells were seeded to the lower chamber. Some lower chambers were left without cells. All incubations were done using medium with 50% HSC-3 medium/50% THP-1 medium with 0.1% BSA instead of serum. Incubations were done for 17 hours and after the incubation the membrane inserts were fixed with 10% TCA, washed with water and air dried. The membranes were then stained with 0.1% crystal violet. Cells attached to the upper side of the membranes were removed by gentle scrubbing. The cells that had migrated to the lower site of the membrane insert were photographed with a Leica DFC 480-camera attached to a Leica DMRB microscope (Leica microsystems, Wetzlar, Germany). The area of the stained cells that had migrated to the lower side of the membrane was further analysed with a Leica Qwin V3 software. Each experimental group was done in triplicate.

Chemotactic cell migration was also tested with the xCELLigence system (Roche Diagnostics AS, Oslo, Norway). 50 000 HSC-3 cells in SF medium with 0.1% BSA were seeded to the upper inserts containing electrodes in migration plates. Mfs-CMs were added to the lower chambers and the cells were allowed to migrate for 48 hours. Cell migration was monitored with the xCelligence system detecting changes in impedance over time as cells migrate to the electrodes.

### Myoma organotypic culture

To investigate the effect of Mfs and Mf CM on HSC-3 cell invasion we utilized the previously characterized myoma invasion model which has been found to mimic the human tumor microenvironment well [27]. Briefly, human uterine leiomyoma tissue was obtained during routine surgeries after the informed consent of the donors (see Ethics statement). The myoma discs were cut with an 8 mm biopsy punch and placed into Transwell inserts (diameter 6.5 mm; Corning). HSC-3 cells (400 000 cells) alone or in co-culture with M1, M2 or R848 Mfs (400 000 cells) in 50 μl of normal growth medium were seeded on top of the myoma discs. In another experiment, myomas were pre-incubated with either M1, M2 or R848 Mf CM. The same CM was also used throughout the experiment as incubation medium. To these myomas, HSC-3 cells (400 000 cells) were put on top of the myomas. One set of myomas were also pre-incubated with either 10 μM BAY 11-7082, 50 ng/ml R848 solution or a similar amount of DMSO in SF-medium. These compounds were also added to the incubation medium during the experiment. The next day myomas were transferred onto nylon membranes placed on steel grids and 1 ml of THP-1/HSC-3 mixed medium was added to the wells with or without experimental compounds. In the second experiment, Mf CMs was used. Medium was collected and changed on days 4 and 7 of the culture period which was 10 days long. Each experimental group was done in triplicate. After 10 days of incubation, myomas were cut in half vertically in the middle of the myoma. One half was fixed in formalin for 24 hours and the other half was snap-frozen in liquid nitrogen to be used for biochemical analysis. After formalin fixation myoma halves were processed into paraffin blocks and 5 μm tissue sections were cut from the sectional area. The sections were deparaffinized and stained with Mayer’s hematoxylin&eosin (H&E). Thereafter myoma sections were immunostained for pancytokeratin with AE1/AE3 antibody (Dako AS, Glostrup, Denmark) as previously described [27]. The immunostained sections were photographed with a Leica DFC 480 camera attached to a Leica DMRB microscope using the LAS v3.8 software. Invasion depth and invasion area was analysed using the QWin V3 software as previously described [27]. The areas of immunostained noninvading and invading cells were calculated and the invasion index (1 − [noninvading area/total area]) was calculated as previously described [27], and the maximal invasion depth per microscopic field (the distance of the invaded cell clusters from the lower surface of the non-invasive cell layer) was measured.

### Zymography

The Mf-CM which was used in the myoma organotypic experiment and collected at days 4 and 8 was analysed by gelatin zymography as previously described [27].

### Matrigel invasion assay

The BD Biosciences (CA, USA) BioCoat Tumor Invasion system was utilized for the testing of cell invasion on Matrigel according to the manufacturer’s instructions. In one set of experiments, 10 000 unlabeled Mfs or HSC-3 cells were cultured at the bottom of the lower chambers while Vybrant DiO-labeled HSC-3 cells were cultured in the upper insert in serum-free medium with 0.1% BSA. Cells in the lower chambers were cultured with or without serum. Cells were cultured for up to 96 hours and readings were measured with a Victor X3 plate reader (Perkin Elmer, CA, USA) at 490/535nm Ex/Em at 24, 48 and 96 hours. An identical set of experiments were done with an uncoated otherwise identical reference plate to measure the migration of cells.

### Mouse xenograft model

Myoma or rat tail collagen gels with embedded gingival fibroblasts [63] were transplanted subcutaneously to 11 weeks old Balb-c nude (nu/nu) mice (n = 5 per group, totally 20 mice, see Ethics statement). Mice were fed and given water *ad libitum*. The myomas and collagen gels were pre-cultured either with or without 400 000 HSC-3 cells for 10 days prior to transplantation as described [27]. A PET plastic dome (1 cm in diameter) was carefully placed on top of the myomas and gels prior to transplantation to prevent smearing of the top HSC-3 cell layer during the transplantation procedure. Two groups of nude mice with myomas/collagen gels were left without HSC-3 cells. Mice were sacrificed after 6 weeks. Implanted xenografts were collected in 4% formalin and embedded in paraffin. Xenografts were sectioned and stained with Mayer’s H&E or immunostained for mouse macrophage marker F4/80 (clone A3–1, Abcam, Cambridge, UK). The sections were photographed using a Leica DFC480 camera attached to a Leica DMRB microscope with the LAS v. 3.8 software. The percentage area of Mfs were measured from the macrophage dense interface between gel/myoma and mouse host tissue and macrophage cell counts were calculated from the gel/myoma tissue itself.

### Immunohistochemistry of myomas and myoma/gel transplants

Six μm myoma tissue sections were deparaffinized and subjected to immunohistochemistry as previously described [27]. Briefly, endogenous peroxidase activity was blocked with 0.3% H_2_O_2_ in methanol, followed by microwaving in citrate buffer (Real Target Retrieval Solution, Dako, Glostrup, Denmark). Non-specific antibody binding was inhibited by normal goat/horse serum (Vector Laboratories, Burlingame, CA, USA) in 2% BSA and then incubated with either rat monoclonal F4/80 (clone A3–1, Abcam, Cambridge, UK) or mouse monoclonal AE1/AE3 pancytokeratin (Dako, Glostrup, Denmark) at 37°C for 30 min, followed by incubation at 4°C o/n. The samples were stained using the Vectastain Elite ABC kits with the respective biotinylated secondary antibodies (Vector Laboratories, CA, USA). Visualization was done with 3-amino-9-ethylcarbazole (Vector Laboratories, CA, USA) as chromogen, counterstained with Mayer`s hematoxylin (Sigma Aldrich Co.LLC, MO, USA), and mounted in Glycergel (Dako, Glostrup, Denmark) or Aquamount (BDH Laboratory Supplies, Poole, UK).

For double staining, 5 μm thick paraffin-fixed tissue sections from human tongue carcinomas (see Ethics statement) were deparaffinized, peroxidase blocked with Peroxidase block (Dako, Glostrup, Denmark) and antigen retrieval was done by microwaving in citrate buffer. Non-specific antibody binding was blocked as described above. First, sections were incubated with either polyclonal rabbit NF-κB p50 (1:200, NLS, Santa Cruz Biotechnology, TX, USA) or polyclonal rabbit NF-κB p65 (1:200, Enzo Life Sciences, NY, USA). The antibodies were applied overnight where after horseradish peroxidase-conjugated secondary antibody (1:200 in 0.1%BSA/PBS) was applied to the tissues for 1 h. SG colour (grey/black colour; Vector Laboratories, CA, USA) was used to visualize the antibodies. Thereafter sections were incubated with a second set of antibodies: polyclonal rabbit NF-κB p65 (1:200, Enzo Life Sciences, NY, USA) or monoclonal mouse CD163 (1:200, EDHu-1, AbD Serotec, NC, USA). The second set of antibodies was visualized with AEC (red colour; Vector Laboratories, CA, USA). Finally, the samples were mounted with Aquatex (MerckMillipore, MA, USA. To validate the staining, some tissue sections were treated with non-immune serum (mouse/rabbit, Dako, Glostrup, Denmark). All immunostained tissue sections were photographed using a Leica DFC480 camera attached to a Leica DMRB microscope with the LAS v.3.8 software.

### Immunofluorescence

VybrantCM-Dil labeled HSC-3 cells (3000 cells) with our without Vybrant DiO-labeled M1-, M2- or R848 Mfs (6000 cells) were grown in 8-well TC Lab-Tek Chamber Slides (Thermo Fischer Scientific, MA, USA). Cells were then washed with PBS, fixed with 4% paraformaldehyde for 10 min at +4°C and permeabilized with methanol at -20°C. Non-specific binding was blocked with normal goat/horse serum diluted 1:50 in 2% BSA/PBS for 30 min at RT. Polyclonal rabbit NF-κB p50 (1:400, NLS, Santa Cruz Biotechnology, TX, USA), polyclonal rabbit NF-κB p65 (1:200, Enzo Life Sciences, NY, USA), monoclonal mouse CD163 (1:100, EDHu-1, AbD Serotec, NC, USA), monoclonal mouse CD68/SR-D1 (1:100, R&D Systems, MN, USA) or monoclonal mouse AE1/AE3 pancytokeratin (1:400, Dako, Glostrup, Denmark) in 1% BSA/PBS were applied and kept for 30 min at 37°C followed by o/n at +4°C. Secondary antibodies were diluted 1:200 in 0.1% BSA/PBS and kept for 1 h at RT in dark. After washing samples were mounted with Shandon Immu-Mount with DAPI (Thermo Fisher Scientific, MA, USA). Images were recorded using a Leica TSC SPS confocal laser microscope and the Leica Application Suite Advanced Fluorescence software.

### Ethical statement

The animals study was approved by the Animal Care and Use Committee at the University of Oulu and the National Animal Care and Use Committee of Finland (OLH-2006-02521/Ym-23, OLH-2006–01987/Ym-22, ESLH-2008-09631-Ym-23, ESLH-2008-03956-Ym-23). Patients (oral cancer tissue samples), had signed an informed consent form and data inquiry has been approved by the National Supervisory Authority for Welfare and Health (VALVIRA), #6865/05.01.00.06/2010 (5.10.2010), and the Ethics Committee of the Northern Ostrobothnia Hospital District, statement 49/2010 (16.8.2010). Use of patient material (myomas) for this study was approved by the Northern Ostrobothnia Hospital District Ethics Committee (statement #8/2006, amendment 19/10/2006 and statement #35/2014, 28.4.2014).

### Statistical analysis

The results from the Oris Pro, Transwell, xCelligence migration assays and from the Matrigel invasion assay were analysed statistically by using One-way Anova. The results from the myoma experiment were analyzed statistically by using Mann-Whitney U-Test except invasion index was calculated using One-Way Anova. For multiple comparisons test in the xCelligence migration assay analysis, Tamhane T2-method was used because the variance of the compared groups differed. P-values of less than 0.05 were considered significant. All statistical analyses were performed using SPSS 20-software (SPSS Inc., Chicago, IL, USA).

## Results and Discussion

### HSC-3 carcinoma cells merge with Mfs in co-cultures

Costea *et al*. [18] showed that carcinoma cells and TAMs appear to be in close vicinity in the oral TME. It is not yet fully clear to which extent tumor cells and TAMs interact through cell-cell contact and paracrine signaling *in vivo*. Keller *et al*. [29] showed that in cell culture conditions, pro-inflammatory M1 type Mfs can kill and phagocytose tumor cells. However, studies addressing the direct interaction of M2/TAM type Mfs and tumor cells are lacking. We therefore wanted to study in more detail Mfs of different subtypes in co-culture with tumor cells. THP-1 cells were polarized to M1, M2 and R848 Mf subtypes. When cell density of Mfs grown without HSC-3 cells was optically measured, M1 Mfs slightly increased in number up to day 7 while M2 and R848 Mfs showed survival up to day 4 after which cell density decreased (S1 Fig.). This indicates that in long-term cultures, M1 Mfs are viable for a longer period of time compared to M2 and R848 Mfs.

**Fig 1:**
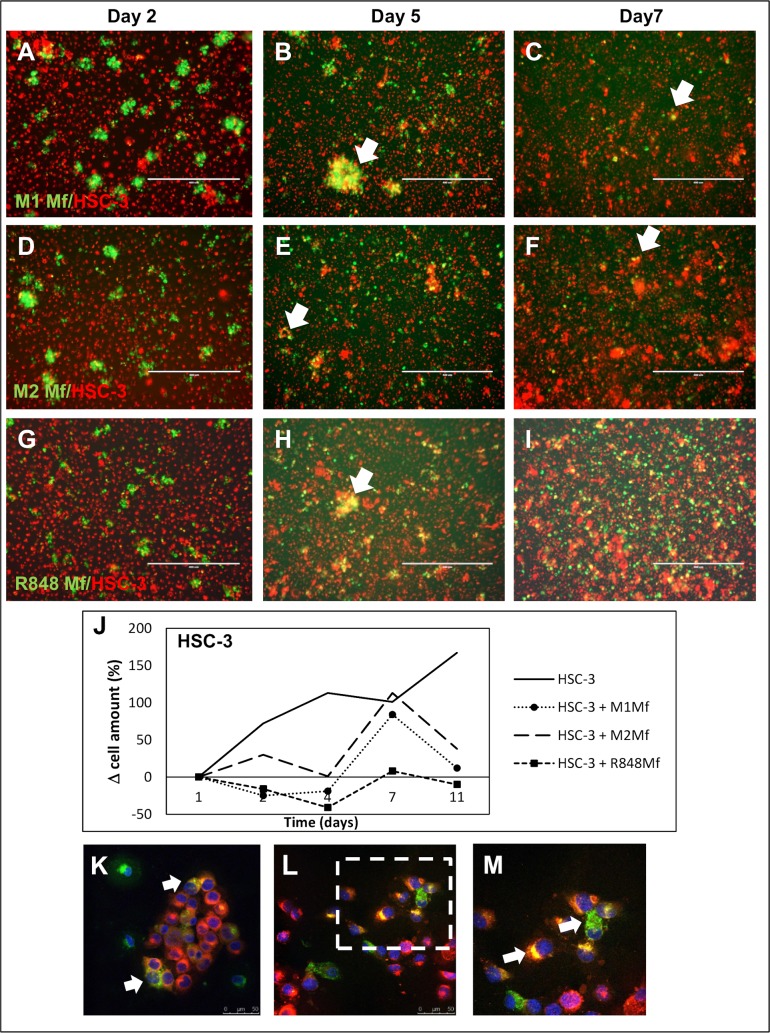
Co-culture of HSC-3 cells and Mfs. Vybrant CM-Dil labeled HSC-3 (red) and Vybrant DiO labeled Mfs (green) were co-cultured for up to 11 days in normal growth medium and photographed with an Evos FL Cell Imaging System microscope. Cell density was analyzed optically using Leica QWin3 Software. Co-culture of labeled HSC-3 and M1 Mfs (A-C), HSC-3 and M2 Mfs (D-F) and HSC-3 and R848 Mfs (G-I) at days 2, 5 and 7. White arrows shows merged/fused cells. (J) Relative HSC-3 cell density in co-cultures. Scale bars 400 μm and n = 2. Representative confocal microscope pictures of co-cultured HSC-3 and R848 Mfs are shown in K-M. Fusion of membranes was observed between the two cell types (white arrows). An R848 macrophage appears to deliver parts to surrounding HSC-3 cells (L), larger picture in M. Scale bars 50 μm in K,L and 25 μm in M.

Thereafter, we co-cultured labeled HSC-3 tongue carcinoma cells (red) with labeled THP-1 Mfs (green). We first established that HSC-3 cell proliferation was not affected by any secreted factor from any of the used Mf subtypes and that HSC-3 cells proliferated normally (not shown). At day 2, the Mfs formed islands within the more uniform HSC-3 layer (Fig. 1A,D,G). At day 5, co-localization of HSC-3 and Mfs could be seen (Fig. 1B,E,H white arrows) as yellow merging. Whether this was fusion of HSC-3 and Mfs or phagocytosis by either cell line could not be established in this experiment. The cells were labeled with Vybrant DilO and CM-Dil (from Life Technologies) respectively which are incorporated into the lipid membranes. Thus, the merging would indicate fusion of membranes.

Interestingly, at days 7–10 of co-cultures, the amount of M1 (Fig. 1C) and M2 Mfs (Fig. 1F) had diminished in the co-cultures while the amount of R848 Mfs was still abundant (Fig. 1I). Also, while only few dispersed fused cells could be seen in the HSC-3/M1 (Fig. 1C) and HSC-3/M2 Mfs co-cultures at days 7–10 (Fig. 1F), clusters of fused cells were abundant in R848 Mfs and HSC-3 co-cultures (Fig. 1I). When co-cultures were optically analyzed, we found that over time, the amount of M1 and M2 Mfs in co-cultures was similar to that found in cultures without HSC-3 cells (S1 Fig.). In contrast, the amount of R848 Mfs in co-cultures increased up to day 7 after which the amount dropped (S1 Fig.). When the amount of HSC-3 cells in co-cultures was analyzed, the amount was growing up to day 7 after which it diminished in contrast to the HSC-3 cultures without Mfs where HSC-3 cells continuously proliferated even after 14 days (Fig. 1J). A reduction in the amount of HSC-3 cells appeared at day 7 simultaneously with the drop in macrophage amount. Interestingly, while R848 Mfs appeared to benefit from the co-culture with HSC-3 in comparison to being cultured alone, HSC-3 cell proliferation on the other hand was readily reduced by co-culture with R848 Mfs. This indicates that cell-cell contact between R848 Mfs and HSC-3 cells can reduce the amount of tumor cells without affecting the Mfs themselves. The polarization of monocytes to tumor-reducing-macrophages may be one way in which Imiquimod reduces tumor growth.

Co-cultures were further analyzed by confocal microscopy by which HSC-3 cells and Mfs were found in close vicinity prior to merging. Fusion of plasma membranes could clearly be observed (Fig. 1K-M). In a further closer view, macrophage cell content appeared to be incorporated into HSC-3 cells that changed color from red to yellow (Fig. 1K-M).

### Merging of HSC-3 cells and Mfs is partially due to efferocytosis

To evaluate if the observed fusion of HSC-3 cells and Mfs was due to efferocytosis, a process where dying cells are removed by phagocytic cells (usually Mfs), we treated the cells with an inhibitor for efferocytosis/macropinocytosis, amiloride (AMI) [30]. Based on previous publications, the effect of AMI is concentration dependent and we therefore tested 0.3 and 3 mM concentrations [30]. In addition, NF-κB is known to regulate TAMs and cancer-related processes and thus, we also tested the effect of NF-κB inhibitor BAY 11–7082 on co-cultures. We first tested the compounds on the cell lines separately. Dimethylsulfoxide (DMSO; used as vehicle) had no effect on the cells cultured alone. At day 2 both HSC-3 and Mfs were slightly rounded by BAY 11-7082 treatment while AMI at 3 mM induced HSC-3 cells to form elongated connections between neighboring cells forming a network-like organization of cells (not shown).

**Fig 2:**
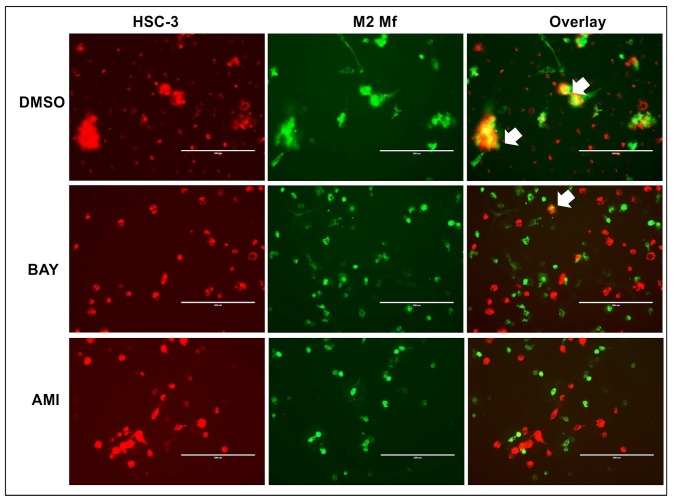
Efferocytosis in HSC-3 and Mf co-cultures. Vybrant CM-Dil labeled HSC-3 (red) and Vybrant DiO-labeled M2 Mfs (green) were co-cultured, treated with 3 mM efferocytosis-inhibitor amiloride (AMI) or 10 μM NF-κB inhibitor BAY 11-7082. Co-cultures of HSC-3 cells and M2 Mfs were photographed with an EVOS FL Cell Imaging System microscope. Pictures presented are from day 2 (n = 4). Fusion of membranes was observed between HSC-3 cells and M2 Mfs in DMSO (vehicle)- and BAY-treated cultures (white arrows in Overlay-section). Scale bars 200 μm.

In DMSO-treated co-cultures at day 2, merging of Mfs and HSC-3 cells was visible as expected (Fig. 2, only HSC-3/M2 Mf co-cultures are shown). Merging was also observed in BAY 11–708 (Fig. 2) and 0.3 mM AMI (not shown) treated co-cultures although to a lesser extent. Fusion cells were almost absent in cultures treated with 3 mM AMI (Fig. 2). Thus, we concluded that the observed merging of Mfs and HSC-3 cells was at least partially due to efferocytosis. There is strong evidence that Mfs can fuse with tumor cells to form heterotypic giant cells and these fusion cells have been indicated in metastatic processes [31]. The heterotypic fusion cells have historically been difficult to identify in individuals but there are a few cases that have been verified [32]. Experiments with fusion cells has shown that typical characteristics are increased metastatic potential, increased sensitivity to pro-migratory signals and increased resistance to chemo- and radiotherapy [33]. Fusion might arise through aberrant phagocytosis of apoptotic tumor cells by Mfs [34]. This could very well be the case in our study, since at least some of the fusion of HSC-3 cells and Mfs was inhibited by AMI. It has also been suggested that the fusion is a way of tumor cells to transfer pro-tumorigenic material to surrounding stromal cells and this has been partially shown *in vitro* [34].

### Co-culture of HSC-3 cells and Mfs alter the cytokine content *in vitro*

Different types of Mfs are known to produce specific cytokines while there is no comprehensive cytokine profile of HSC-3 cells. We hypothesized that co-culturing HSC-3 cells and Mfs would alter the cytokine profiles. Therefore, SF-media from HSC-3 cells, the three Mf subtypes and the three co-cultures (HSC-3 with M1-, M2 or R848 Mfs) at day 2 were collected and analyzed for 80 cytokines with the RayBio Human Cytokine Antibody Array. The array values were normalized against positive control, background and sample protein content. The analysis showed that HSC-3 cells contained low levels of all the analyzed cytokines as compared to the Mfs with a few exceptions. Therefore, the antibody array values for HSC-3 were chosen for calculation of fold change in comparison to the other groups. Cytokines with inter-group fold changes of at least 2x were considered of interest. Of all groups, the M1 Mfs cultured alone showed the highest levels of all cytokines. For representative presentation, cytokines with similar fold changes (e.g. max fold change 25x in figure A etc.) were grouped into the same graphs. The full antibody array data is provided in the supporting information (S1 Dataset).

**Fig 3:**
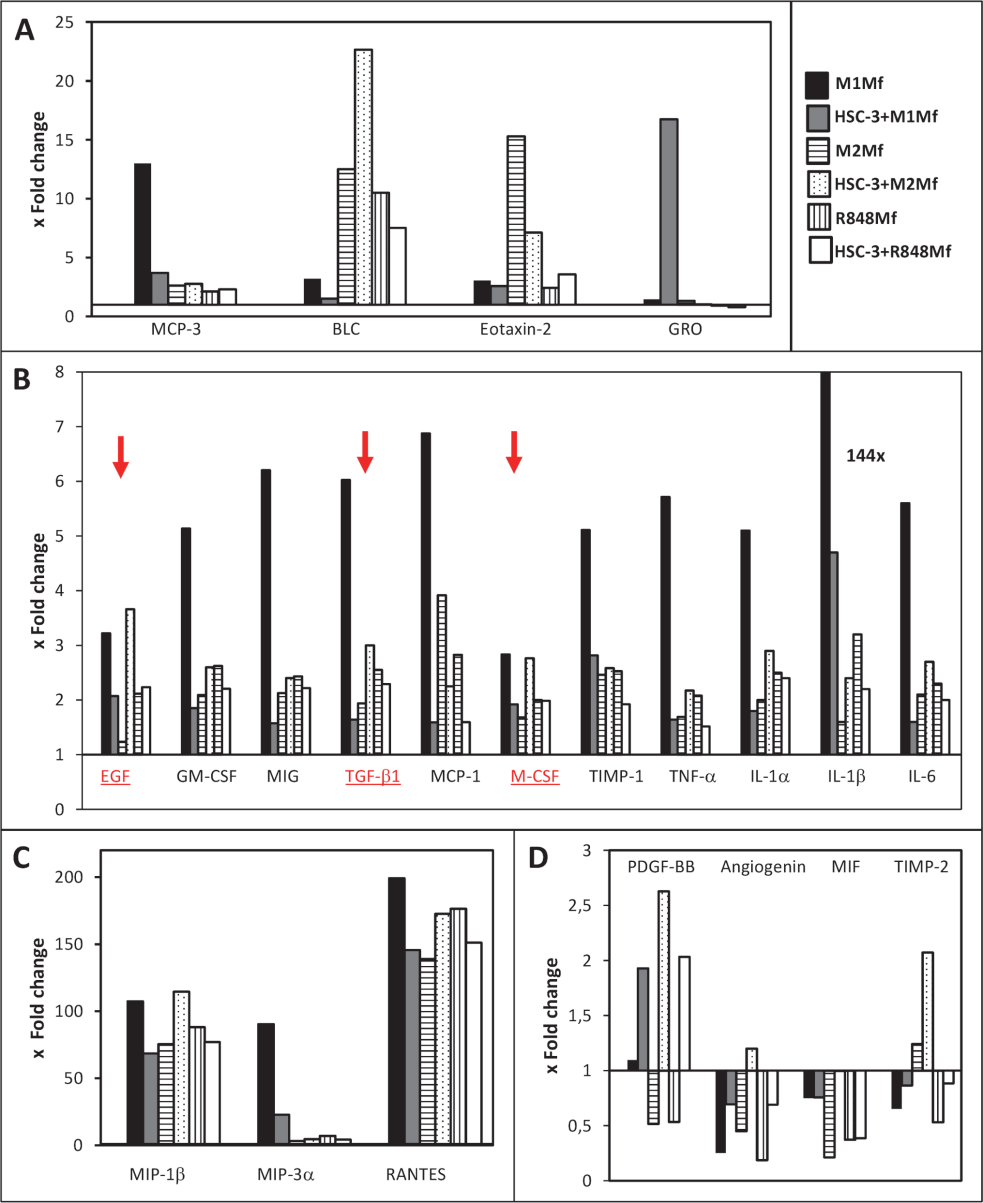
Cytokine production in HSC-3 cells and macrophage co-cultures. Serum-free media from HSC-3 cells, Mfs and co-cultures were collected at day 2 and analyzed for cytokines with RayBio Human Cytokine Antibody Array G5. The values for relative fluorescence intensity were normalized and background was reduced. Thereafter the values were normalized against sample protein content. HSC-3 cells contained low levels of almost all cytokines (with a few exceptions) as compared to the Mfs and the antibody array values for HSC-3 were chosen for calculation of fold change in comparison to the other groups. Cytokine data in the figure are grouped and presented based on mean fold change levels: up to 25x (A), up to 8x (B) and up to 200x (C). Four cytokines had fold changes that were higher in HSC-3 cells than in Mfs (D). EGF, TGF-β1 and M-CSF are marked in red with red arrows.

M1 Mfs medium contained higher amounts of TNF-α, Granulocyte—Macrophage Colony-Stimulating Factor (GM-CSF), Monokine Induced by Gamma Interferon (MIG), Tissue Inhibitor of Metalloproteinases (TIMP)-1 (Fig. 3B) and Monocyte Chemoattractant Protein-3 (MCP-3) (Fig. 3A) than the other groups. Co-culture of M1 Mfs and HSC-3 cells reduced the secretion of these cytokines several-fold down to levels seen in the other groups (Fig. 3A,B). TNF-α has been shown to be the major inflammatory signal for macrophage lytic activity against tumor cells [29]. However, TNF-α is also associated with sustained chronic inflammation of the TME through NF-κB regulation [35]. High levels of TNF-α have been found in head and neck (HNSCC) patient serum and in malignant oral carcinoma cell lines [36]. Thus, while TNF-α, MCP-3 and GM-CSF are pro-tumorigenic, we found that co-culture with anti-inflammatory M1 Mfs and HSC-3 cells actually down-regulated these cytokines.

MIG on the other hand is involved in T cell trafficking and has been found to be anti-cancerous and also previously found in high amounts in M1 Mfs [37]. Interestingly, a previous study found that NF-κB mediated polarization of M2 Mfs to M1 Mfs was associated with an increase in MIG secretion [38]. In addition, TIMP-1 is a potent inhibitor of several matrix metalloproteinases (MMPs), including MMP-1, -8 and -9. Thus, down-regulation of MIG and TIMP-1 in HSC-3 cells and M1 Mfs is in favour of pro-tumoral processes.

Transforming Growth Factor (TGF)-β, Epidermal Growth Factor (EGF) and Macrophage Colony-Stimulating Factor (M-CSF) (Fig. 3B) were all found in higher amounts in M1 Mfs while the levels were slightly lower in M2 and R848 Mfs. However, co-culturing M1 Mfs and HSC-3 resulted in several-fold lower levels of all these cytokines while M2 Mfs and HSC-3 co-cultures resulted in higher levels as compared to the M2 Mfs alone (Fig. 3B). TGF-β is associated with M2 type Mfs and tumor cell proliferation, tissue fibrosis, immunosuppressive activity and epithelial-to-mesenchymal-transformation [23]. Specifically, TGF-β promotes fibroblast-to-CAF trans-differentiation and accumulation of CAFs is significantly correlated to poor prognosis in tongue cancer [8]. In this regard, high levels of TGF-β have been found in OSCC TAMs and has been associated with metastasis [18]. EGF is a growth factor highly associated with progression of OSCC [36] and is intricately associated with M-CSF, a cytokine involved in macrophage recruitment and differentiation [39]. Similar to our results, Yang *et al*. [40] found that M-CSF production was increased in Mfs of mouse breast carcinoma cells and Mfs co-cultures inducing polarization from M1 to M2 type Mfs through NF-κB p50 induced c-Jun expression. *In vivo*, M-CSF produced by carcinoma cells attracts TAMs to nearby blood vessels which in turn promote the expression of EGF by Mfs [33]. In our study, M-CSF was produced by HSC-3 cells although to a lower extent than in the Mfs. The EGF in turn attracts tumor cells to the blood vessels and a positive feedback loop is created, feeding the growth and local invasion of tumor cells [33,36]. Clearly, co-culture of HSC-3 and M2 Mfs in this study induced expression of both EGF and M-CSF as well as TGF-β further underlining the important role for these three cytokines in oral cancer progression (Fig. 3B, underlined and indicated with red color).

All groups showed a similar fold change of all the studied interleukins with little inter-group variations, except for the M1 Mfs cultured alone which showed a higher fold change for all interleukins and a 144x fold change for IL-1β (Fig. 3B). Interestingly, co-culturing M2 or R848 Mfs with HSC-3 did not particularly affect interleukin levels as compared to Mfs cultured alone. However, incubating M1 Mfs with HSC-3 cells consequently reduced interleukin secretion several-fold as compared to the M1 Mfs alone (Fig. 3B). A study by Engström *et al*. [37] showed very similar results in terms of interleukins secreted by M1 Mfs, further re-enforcing our results. IL-1β, secreted to a high amount by the M1 Mfs is pro-inflammatory, however it has also been found to participate in trans-differentation of fibroblasts-to-CAFs [41].

The highest fold-changes (up to 200x) were found for Macrophage inflammatory protein (MIP)-1β, MIP-3α and RANTES (Fig. 3C). When M1 Mfs were co-cultured with HSC-3 cells, the levels were reduced and conversely, when M2 Mfs were incubated with HSC-3 cells the levels increased (Fig. 3C). We found low amounts of MIP-3α and RANTES in HSC-3 cells in concordance with previous publications [42,43] and also MIP-1β (not shown) which has not previously been described in OSCC cell lines. Previous work has confirmed that RANTES, MIP-1α and MIP-1β and their receptor C-C Chemokine Receptor type 5 (CCR5) are involved in the recruitment of M1 Mfs to tumor sites [39,44]. Dysregulated CCR5 expression on Mfs have been found in OTSCC patients which may contribute to reduced anti-tumoricidal activity [44].

The only cytokines that were expressed to a higher level in HSC-3 than in some Mfs were Plateled-derived growth factor (PDGF)-BB, angiogenin, Macrophage migration Inhibitory Factor (MIF) and TIMP-2 (Fig. 3D). In addition, the fold change of all four cytokines increased in co-cultures of HSC-3 /Mfs as compared to Mfs alone (Fig. 3D). Circulating tumor/macrophage hybrid cells have been found to release MIF through exosomes which then creates metastatic niches [32]. MIF has previously been found to be secreted by M2 Mfs [33] but we found that HSC-3 cells secreted more MIF than M2 Mfs. Yaddanapudi *et al*. [45] showed that MIF controls activation of M2 Mfs in melanoma tumor-carrying mice. The role of MIF in OSCC progression is controversial [46,47] but inhibiting MIF activity in OSCC cells has been found to inhibit invasion and proliferation [46,48]. TIMP-2 expression has been found in tumor cells at the invasive front as well as in the stromal cells [49]. TIMPs expression in OSCC is mostly associated with poor outcome [50] and our results corroborate well with this. Interestingly, while HSC-3 cells secreted more PDGF-BB than the Mfs, co-culture increased secretion regardless of macrophage subtype. PDGF is involved in the modulation of the TME [51] and a recent study showed that TAMs expressed PDGF-BB only when they adhered to cancer stem cell-like breast cancer cells and this was modulated through hyaluronan synthase [52].

Taken together, we found that cytokines shown to be associated with OSCC and increased invasion, such as EGF, M-CSF, TGF-β, TIMPs, PDGF-BB and MIF were increased upon co-culture with HSC-3 and M2 Mfs, and on the other hand down-regulated in HSC-3/M1 Mfs co-cultures. Thus, these cytokines are well suited for further investigation as therapeutic targets in OSCC.

### An M2 Mf-secreted soluble factor induces migration of HSC-3 tongue carcinoma cells

We continued with investigating the effect of Mfs on HSC-3 cell migration, something which has not previously been characterized. We used different migration systems to investigate the effect of Mfs on HSC-3 cell migration.

First, we tested the effect of macrophage-HSC-3 cell-cell contact on HSC-3 cell migration in a horizontal plane using the Oris Pro cell migration system. In this system, cells are grown in a 96-well plate around an insert which is then removed, allowing cells to migrate to the empty space.

**Fig 4:**
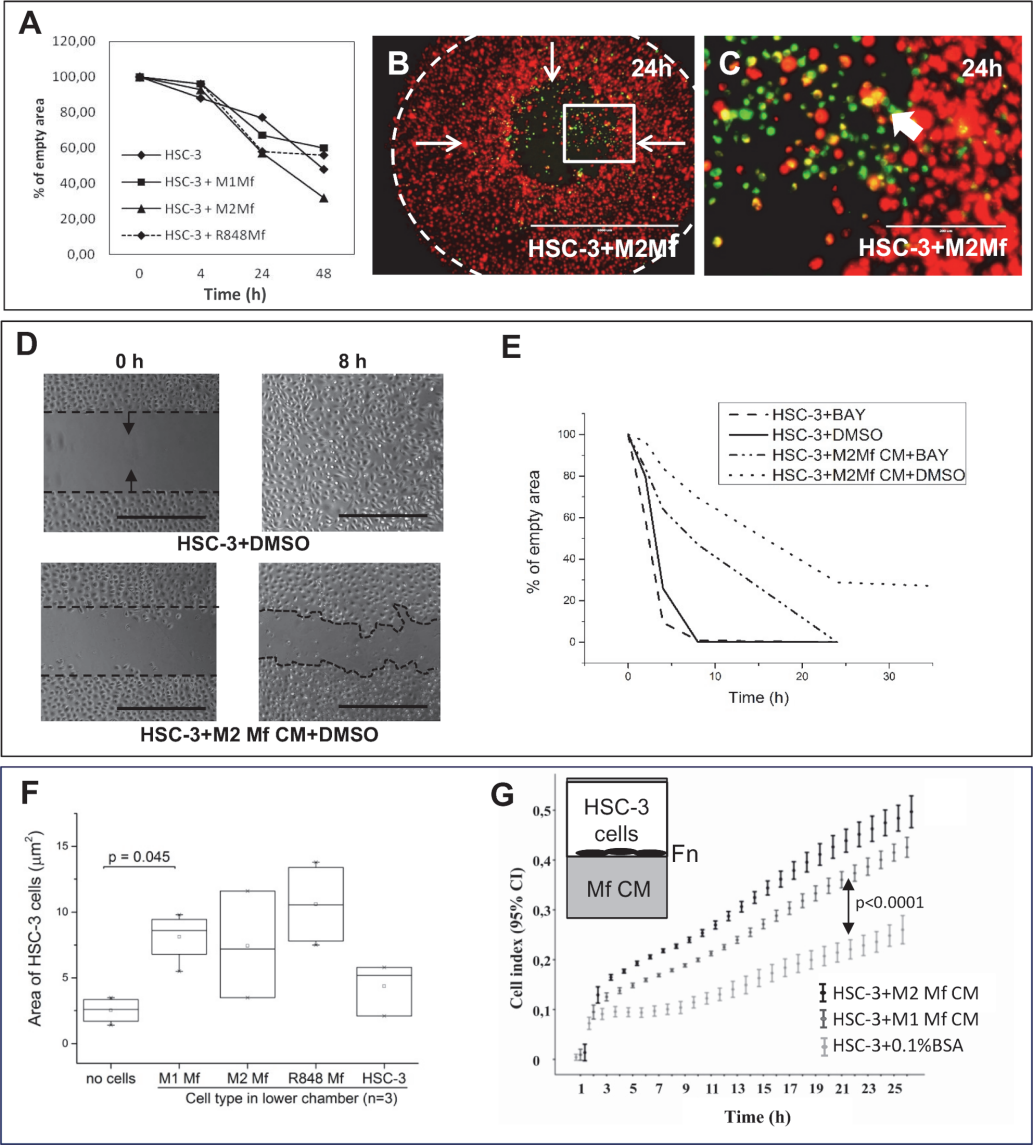
The effect of Mfs, Mf-CM and NF-κB inhibition on HSC-3 cell migration. Horizontal migration: (A-C) Vybrant CM-Dil labeled HSC-3 (red) and Vybrant DiO-labeled Mfs (green) were co-cultured in Oris Pro cell migration 96-plates. Inserts were then removed and cells allowed to migrate into the empty space. (D-E) HSC-3 cells were allowed to attach to both sides of Ibidi inserts in culture wells before inserts were removed, Mf CM was added in addition to 10 μM NF-κB inhibitor BAY 11-7082, where after cells were allowed to migrate into the empty space. Migration was photographed with EVOS FL Cell Imaging System microscope over time. The empty space area was measured using Leica QWin3 Software (A) or with the ImageJ-software (E). Representative pictures of HSC-3 and M2 Mfs migration at 24 h (B, C). White arrows show the direction of migration and the dotted line indicates the empty space at time 0h (B). Magnification from the migratory front in B (white box) shown in C (white arrows indicate merged cells). Scale bar in B: 1000 μm and in C: 200 μm. n = 6 for each group. (D) Actual photographs of HSC-3 cell migration treated with M2 Mf CM at 0 and 8 hours (D). Dotted black line shows start point for migration and arrows direction of migration. Scale bars: 500 μm and n = 4. Chemotactic migration: (F) HSC-3 cells were seeded to the upper chamber of Transwell inserts and Mfs were cultured in the lower chamber (n = 3). Migrated cells were stained with Crystal violet and analyzed with Leica QWin3 software. (G) HSC-3 cells were seeded to fibronectin (Fn)-coated xCelligence inserts and Mf-CM was added to the lower chambers (n = 4). Migration was monitored for up to 24 hours. The xCelligence system measures changes in cell impedance as cells migrate through the insert membrane and data is presented as cell index.

In homogenic cell cultures Mfs migrated to the empty space and filled it to approximately 40% at 48 hours while HSC-3 cells had filled the space almost completely at 24 hours with minor changes at 48 hours (not shown). In co-cultures, HSC-3 cells (red) were found to migrate more efficiently in co-culture with M2 Mfs (Fig. 4B,C; Mfs in green), although not statistically significant (Fig. 4A). HSC-3 cells migrated slightly slower in co-culture with M1 Mfs (S1 Fig. and Fig. 4A) and similarly with and without R848 Mfs (S1 Fig. and Fig. 4A). When the migratory front was observed more closely, co-migration together with merging of Mfs and HSC-3 cells could be seen with all Mfs subtypes (Fig. 4B,C and S1 Fig.), white arrows show merged cells). The Mfs appeared to migrate slightly in front of the HSC-3 cells. In co-cultures with M1 Mfs and HSC-3 cells, Mfs were more abundant at 48 hours (S1 Fig.) which could partially explain the reduced migration of HSC-3 cells. These results indicate that when cell-cell contact is propagated in a horizontal plane, M2 Mfs induce migration of HSC-3 cells while M1 Mfs slightly inhibit migration.

We then tested the effect of Mf-conditioned medium (CM) on horizontal HSC-3 migration using Ibidi cell culture inserts. Cells were allowed to attach overnight on both sides of the inserts and thereafter inserts were removed, medium changed to Mf-CM with or without BAY 11-7082 and cells allowed to migrate into the empty space. In this experimental setting, HSC-3 cells treated with Mf-CM migrated slower than DMSO-treated cells (Fig. 4D,E, only M2 Mf-CM is shown). While no statistical significance could be reached, it appeared that M2 Mf-CM had the least effect and M1 Mf-CM slowed HSC-3 cells down more effectively. Interestingly, while BAY 11-7082 did not affect HSC-3 cell migration, addition of BAY 11-7082 slightly reversed the Mf-CM inhibition of HSC-3 cell migration (Fig. 4E). This indicates that the migration inhibitory factor(s) present in Mf-CM is/are partially NF-κB-dependent.

Next, chemotactic migration using the Transwell culture inserts was assessed. HSC-3 cells were cultured in the upper inserts and Mfs in the lower chambers. All Mfs induced migration of HSC-3 cells to a similar efficacy although only M1 Mfs showed statistical significance (Fig. 4F). For comparison, HSC-3 cells were also cultured to the lower chamber of some wells and did not induce migration of the HSC-3 cells in the upper chamber (Fig. 4F). This indicates that the migration inducing factor is specifically produced by Mfs. We also tested if HSC-3 cells were able to attract Mfs, an event that would be critical *in vivo*. Here, HSC-3 cells were seeded to the lower chamber in Transwell-plates while Mfs were seeded to the upper chamber in SF-medium. We found that HSC-3 cells induced migration of Mfs, although statistical significance was not reached (S2 Fig.).

The xCelligence migration assay was further used to analyze the effect of Mf-secreted chemotactic factors on HSC-3 migration. HSC-3 cells were seeded to fibronectin-coated upper chambers and Mf-CM was added to the lower chambers. Significantly more HSC-3 cells migrated towards M2 Mf-CM than towards non-CM (Fig. 4G, p<0.0001). Also M1 Mf-CM induced HSC-3 cell migration significantly (Fig. 4G, p<0.0001), however, not as efficiently as M2 Mf-CM. The experiment was repeated on gelatin-coated chambers and by using uncoated chambers with a highly similar result (not shown). BAY 11-7082 was tested in this experimental setting but was not found to affect chemotactic HSC-3 cell migration. Mf-CMs were also not found to induce proliferation of HSC-3 cells as analysed by xCelligence (not shown). Thus, soluble factors secreted by TAM-like Mfs act as chemotactic agents on highly aggressive HSC-3 cells but this mechanism is not NF-κB dependent. As found in the cytokine antibody array, Mf-CMs contain many chemotactic cytokines such as EGF, M-CSF and MIF. This is the first time that the direct effect of Mfs and Mf-CM on tongue SCC cell migration has been demonstrated.

Interestingly, while the Mf-CM clearly contains chemotactic signals for HSC-3 cells, the Mf-CM also contains inhibitory factor(s) acting directly on HSC-3 cells if applied on horizontal migration settings (haptotactic migration). In chemotactic migration, the CM acts from below creating a gradient of migratory factors for HSC-3 cells while in the horizontal migration settings, the medium acts on the top of cells. The mode of action is clearly dependent on tumor cell orientation.

### Mfs induce HSC-3 cell invasion by cell-cell contact

**Fig 5:**
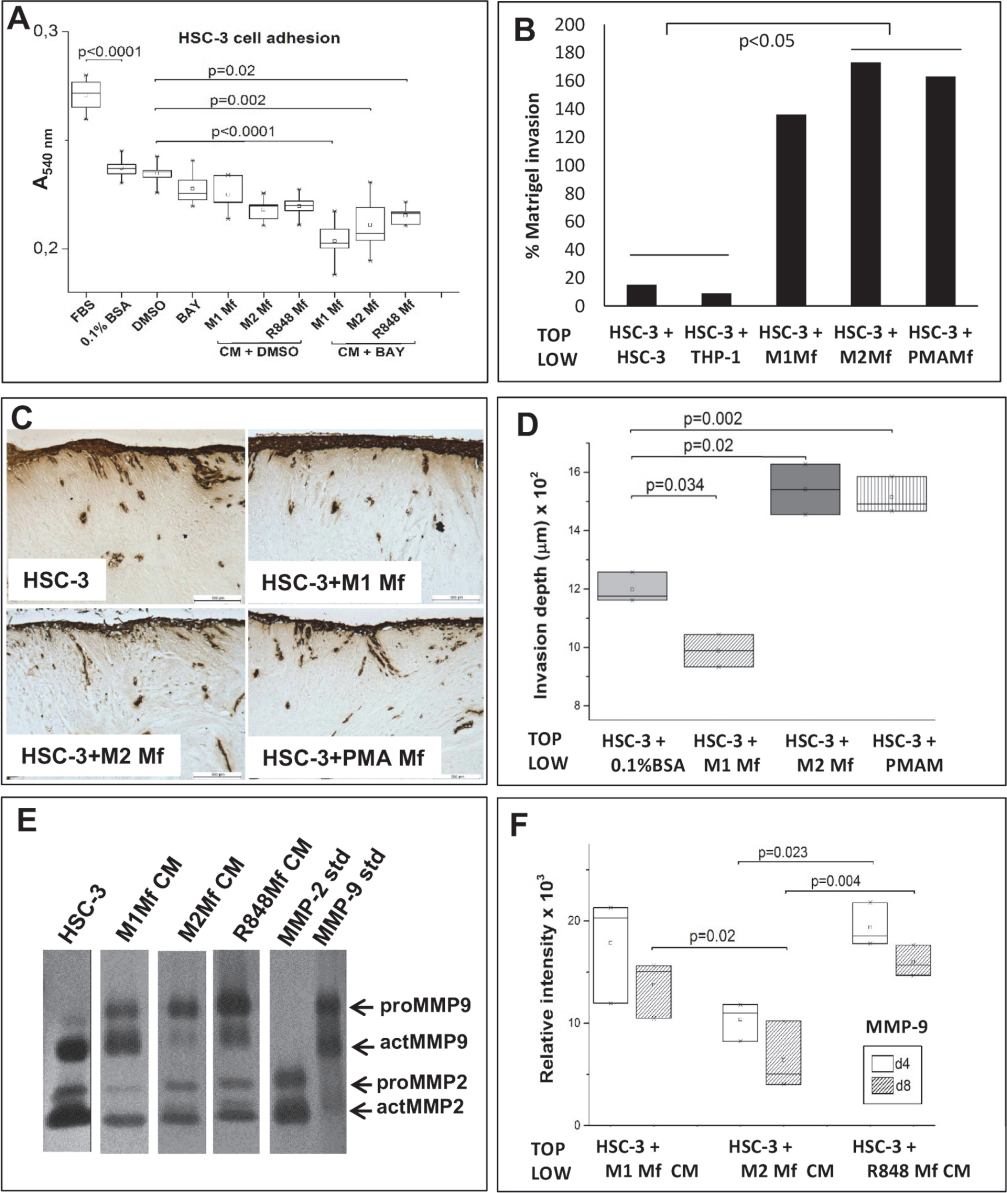
Co-culture of HSC-3 cells and Mfs induce invasion in an organotypic 3D tissue model. (A) HSC-3 cells were treated with Mf-CM and/or 10 μM BAY 11-7082 (n = 6) and adhesion to fibronectin-coated wells was analysed after 2 hours with Crystal violet-staining. Stained wells were analysed by measuring absorbance at 540 nm. (B) Labeled HSC-3 cells were cultured on top of Matrigel and Mfs, HSC-3 or THP-1 cells were cultured in the lower chambers (n = 6). Invaded cells were analyzed from the membrane by measuring fluorescence. HSC-3 cells and Mfs were co-cultured on top of human myoma tissue (C,D, n = 3) for 10 days where after tissues were fixed and processed for immunohistochemistry. Pan-cytokeratin stained sections (in C, scale bars: 500 μm) were photographed and invasion areas and invasion depths were analysed with the Leica Qwin3 software. Conditioned medium was collected at day 4 and 8 from myomas and medium containing 0.5 (Mf CMs) or 15 μg (HSC-3) protein were subjected to gelatin zymography (E, day 4). Samples were done in triplicate, representative picture of samples is shown in E. Recombinant MMP-2 and -9 were used as positive controls. Relative intensity of MMP-9 bands were analyzed with the ImageJ-software (F).

Migration studies only give information about the mobility of cells in a given 2D environment. To further investigate the effect of Mfs on HSC-3 cell mobility, we also studied invasion which requires cells to get through a 3D-barrier. We first tested if Mf-CM had any effect on HSC-3 cell adhesion since this would affect invasion. We found that significantly less HSC-3 cells adhered on fibronectin when incubated in serum free (SF)-medium (supplemented with bovine serum albumin) as compared to HSC-3 cells in medium with fetal bovine serum (Fig. 5A, p<0.0001). DMSO at the same concentration used as vehicle did not affect adhesion as compared to cells in SF-medium with BSA. Cells were then treated with Mf-CM to which BAY 11-7082 or DMSO was added. BAY 11-7082 or M1 Mf-CM alone did not affect adhesion while M2 and R848 Mf-CMs slightly reduced HSC-3 cell adhesion (Fig. 5A, p = 0.07). Only the combination of BAY 11-7082 and Mf-CM significantly reduced HSC-3 cell adhesion as compared to DMSO alone (Fig. 5A, p<0.0001 for M1 Mf-CM+BAY; p = 0.002 for M2 Mf-CM+BAY; p = 0.02 for R848 Mf-CM+BAY). This indicates that while HSC-3 cell adhesion in the absence of Mfs is not NF-κB dependent, Mf-CM (regardless of subtype) contains an NF-κB regulated molecule which promotes HSC-3 cell adhesion.

The strongest effect was seen with M1 Mf-CM, indicating that M1 Mfs activity could be downregulated and NF-κB dysregulated in favour of oral cancer. In a study by Chengye *et al*. [53] peroxisome proliferator-activated receptor gamma-1-related co-activator was found to inhibit expression of adhesion-mediating molecules through inhibition of NF-κB activation.

We then tested HSC-3 invasion using the Transwell-based invasion assay in which HSC-3 cells were cultured on top of Matrigel and the Mfs were seeded to the lower chamber. For comparison, we used also phorbol myristate acetate (PMA) Mfs which are THP-1 cells that have been polarized to Mfs with PMA alone and resemble TAM-like Mfs [54]. As controls we seeded HSC-3 or THP-1 cells in the lower chambers to exclude that any cell type could secrete invasive signals for HSC-3 cells. M2- and PMA-type Mfs significantly induced HSC-3 cell migration compared with controls (Fig. 5B, p<0.05). In this setting, also M1-type Mfs induced invasion of HSC-3 cells although not statistically significant (Fig. 5B).

We then used the fully human 3D hypoxic myoma-based model [27,28] where HSC-3 cells and Mfs were first co-cultured on top of the myoma. After 10 days, the myomas were fixed and further analysed by histology and semi-quantitative computer analysis. We found that both M2- and PMA Mfs significantly induced invasion of HSC-3 cells in myoma tissue (Fig. 5C,D, p = 0.02 and 0.002 respectively) compared to HSC-3 cells alone (Fig. 5C,D). The M1-type Mfs on the other hand significantly reduced HSC-3 cell migration (Fig. 5C,D, p = 0.034). The invasion index was calculated and results were comparable for the invasion depth (not shown). Thus, M2 Mfs act pro-invasive on HSC-3 cells while M1 Mfs inhibits invasion. In another experiment, we also tested the ability of R848 Mfs to induce invasion of HSC-3 in myomas in addition to NF-κB inhibitor BAY 11-7082 and Imiquimod-solution (R848). There were no significant differences in HSC-3 invasion depth between the groups (S2 Fig.). When the invasion index was calculated, R848 Mfs were found to decrease invasion of HSC-3 cells, as did also BAY 11-7082, but the effect was not statistically significant (S2 Fig.). In a second myoma experiment, HSC-3 cells were cultured on top of the myoma and myomas were incubated with Mf-CM. As opposed to the results from migration assays, Mf-CM did not induce invasion of HSC-3 cells (S2 Fig.). This is an important finding showing that although Mf-CM contains pro-migratory signals for HSC-3 cells, it does not necessary induce invasion. Taken together, for the first time we show that the close vicinity of Mfs to oral cancer cells is crucial for both the induction and inhibition of tumor cell invasion through tissues. In addition, R848 Mfs do not appear to affect invasion or migration substantially. However, as observed in the initial co-culture experiments, cell-cell contact with R848 Mfs appears to reduce HSC-3 cell proliferation (Fig. 1J) which might be one of the mechanisms by which these artificial Mfs work.

Medium from the myoma experiment performed with the Mf-CM was collected on days 4 and 8 and analysed by gelatin zymography. We have shown in previous publications that medium collected from cell-free myomas contain only minor amounts MMP-9 but culturing HSC-3 cells on top of myomas results in secretion of MMP-9 into the incubation medium [27]. Also here, SF-medium from the myoma with HSC-3 cells on top contained both MMP-2 and-9 (Fig. 5E, day 4). The uncropped zymograms can be viewed in the supporting information (S3 Fig.).

Mf-CM from the myomas incubated with HSC-3 cells was found to contain high levels of active MMP-9 (Fig. 5E,F). Interestingly, M2 Mf-CM contained the least MMP-9 and the MMP-9 level also decreased between day 4 and 8 (Fig. 5F). In contrast, M1 Mf-CM (p = 0.055 at day 4 and p = 0.020 at day 8) and R848 Mf-CM (p = 0.023 at day 4 and p = 0.004 at day 8) contained significantly more MMP-9 than M2 Mf-CM (Fig. 5F). In addition, in myomas incubated with HSC-3 cells and M2 Mf-CM, MMP-9 was present mostly in the pro-form while in myomas incubated with HSC-3 cells and M1 Mf-CM or R848 Mf-CM, MMP-9 was present both in the active and pro-form. MMP-2 was present in all myoma incubation media and there were no major differences between the groups (not shown). We have recently shown that in HSC-3 cells, MMP-9 actually might have a protective role (Väyrynen *et al*. manuscript in preparation). In this regard, it is interesting to note that when HSC-3 cells are invading tissue in the presence of M1 Mf-CM and R848 Mf-CM, the MMP-9 level remains high, while in the presence of M2 Mf-CM, the MMP-9 level is actually decreasing over time. Zajac *et al*. [55] found that M2 Mfs induced angiogenesis which was mediated through production of high MMP-9 secretion. Similar to our findings, they also found that M1 Mfs produced high levels of MMP-9. However, M1 Mfs also produced high levels of TIMP-1 while M2 Mfs did not and thus, only M2 Mf-produced MMP-9 activated angiogenesis. In parallel with our results, M1 Mfs produced significantly more TIMP-1 as compared to the M2 Mfs. Interestingly, co-culture with HSC-3 cells reduced TIMP-1 production also in M1 Mfs. In this regard, Koontongkaew *et al*. [56] found that direct contact between HNSCC cells and fibroblasts activated MMP-9.

### Modulation of NF-κB in HSC-3 and macrophage co-cultures

The activity of the nuclear transcription factor complex NF-κB is associated with oral carcinomas and is known to be dysregulated in TAMs. The findings in the migration experiments that Mf-CM contains NF-κB regulated molecules acting inhibitory on HSC-3 cell migration lead us to investigate the previously uncharacterized expression and activity of NF-κB subunits p50 and p65 in HSC-3 and macrophage co-cultures.For this purpose, we performed immunofluorescence on cultured HSC-3 and Mfs analyzed by confocal microscopy. All samples were subject to immunostaining with polyclonal and monoclonal non-specific immunoglobulins and showed no unspecific staining (not shown).

**Fig 6:**
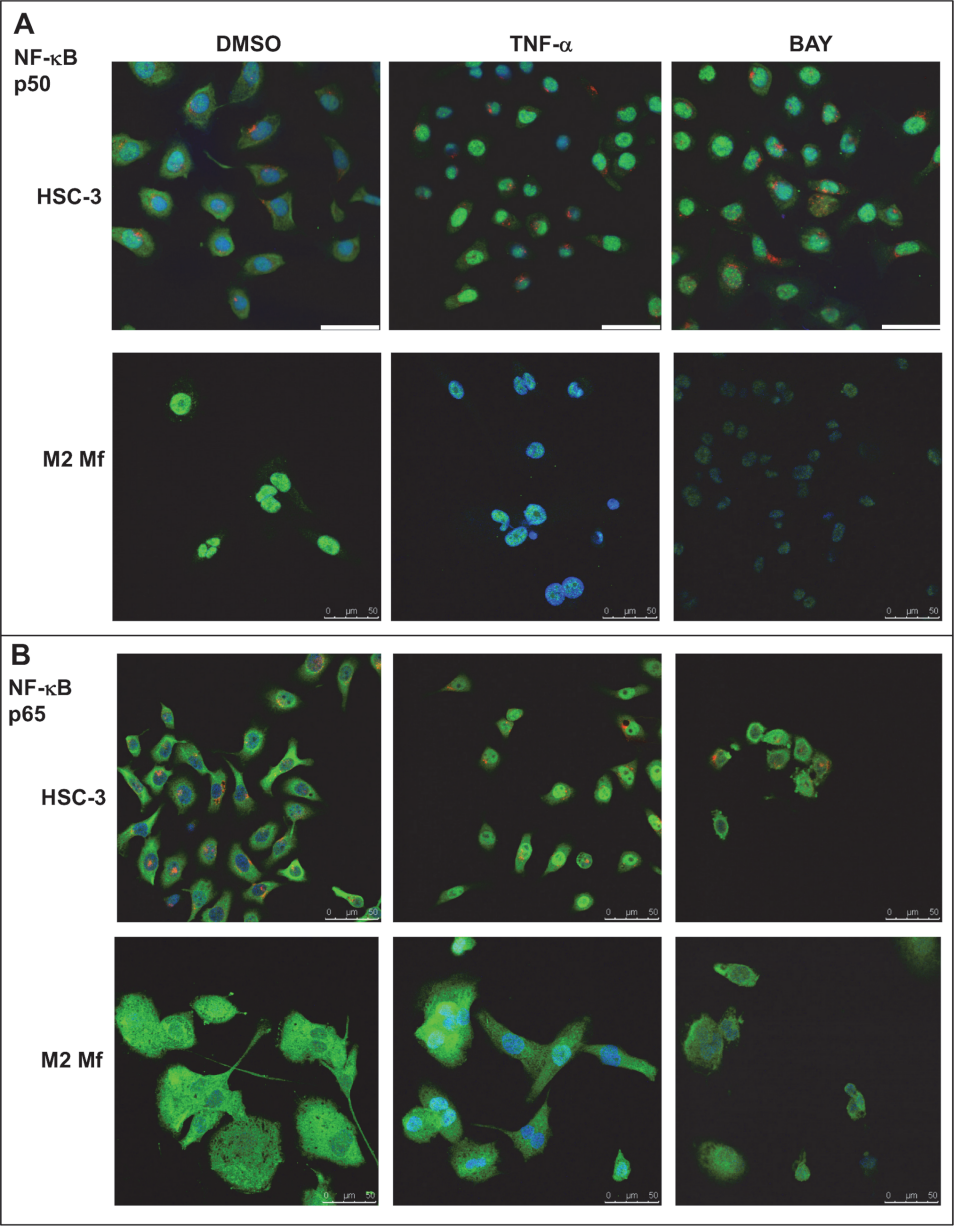
Expression of NF-κB p50 and p65 subunit in HSC-3 and M2 Mfs. Vybrant CM-Dil labeled HSC-3 cells (red) and unlabeled M2 Mfs were incubated with DMSO or 10 ng/ml TNF-α for 30 min where after cells were fixed for immunofluorescence with polyclonal NF-κB p50 (A) or NF-κB p65 (B) antibodies. Some samples were pre-incubated with 10 μM BAY 11-7082 prior to TNF-α activation. AlexaFluor488-conjugated secondary antibody was used for visualization. Samples were mounted with DAPI- mountain medium to visualize nuclei (blue). Samples were photographed with a Leica Confocal microscope with 63x oil immersion objective. Scale bars 50 μm.

M1 Mfs were CD68 positive (S4 Fig.) and CD163 negative (marker for TAM/M2, not shown) while M2 Mfs were positive for both CD68 and CD163 (S4 Fig.). R848 Mfs were CD68 positive (S4 Fig.) but some cells were also CD163 positive (S4 Fig.). HSC-3 cells cultured alone stained positive for pan-cytokeratin (S4 Fig.) and were negative for macrophage markers CD68 or CD163. Both the NF-κB p50 (Fig. 6A) and NF-κB 65 (Fig. 6B) subunits were found in the cytoplasm of HSC-3 cells and TNF-α treatment induced translocation to the nucleus (Fig. 6A,B). BAY 11-7082 mostly but not entirely inhibited nuclear translocation of both subunits. This indicates that the NF-κB activation mechanism itself would be normal in HSC-3 cells. In M2 Mfs, the p50 subunit appeared in the nucleus of resting cells while in the TNF-α treated cells there was less visible p50 in the nuclei. BAY 11–782 reduced the overall expression of p50 (Fig. 6A). Interestingly, the p65 subunit was mostly detected in the cytoplasm of both resting, activated and inhibited M2 Mfs (Fig. 6B). This indicates that there could be an excessive amount of p50 subunits in the M2 type Mfs, perhaps causing a dysregulation of NF-κB activation. In the TME, TAMs are thought to be tolerized by inflammatory stimuli through the formation of repressive p50 homodimers in the nucleus which in turn activate non-tolerable, tumor-progressive favouring genes [23,57]. The formation of p50 homodimers also inhibits the polarization of M1 Mfs [23]. IL-10, prostaglandin E2 and TGF-β, all produced by TAMs and many tumors, induce the formation of p50 homodimers [23]. In this regard, we found that co-culturing HSC-3 cells and M2 Mfs increased TGF-β secretion (Fig. 3B). Connelly *et al*. [38] showed that re-activation of NF-κB signaling reduced tumor burden in mice. While p50 homodimers have been found in complex with bcl-3, acting on gene transcription [58], the p50/p50 complex is mostly thought to repress gene activation. Also, the p50/p50/bcl-3 complex has been found in nasopharyngeal carcinomas but is rare in other HNSCCs [59].

M1 and R848 Mfs were similar in that p50 was present in the nuclei of resting Mfs and TNF-α treatment did not change this appearance as expected (S5 Fig.). The NF-κB p65 subunit on the other hand was similarly present in the cytoplasm of both resting M1 and R848 Mfs, however in the TNF-α activated cells, a nuclear translocation of p65 could be observed in the M1 but curiously not in the R848 Mfs (S5 Fig.). BAY 11–782 treatment decreased the overall appearance of p50 and p65 and also the nuclear translocation in both cell types (S5 Fig.). Thus, the NF-κB activation mechanism present in R848 Mfs is different from both the M1 and M2 Mfs. R848 is a TLR7/8 agonist and is thus expected to induce NF-κB through the MyD88- NF-κB pathway [24]. Imiquimod has so far been used for the treatment of skin carcinomas through the modulation of immune cells and polarization of Mfs in the TME [24,25]. Recently, mucoadhesive films with imiquimods for the treatment of OSCC have been developed [26]. Our results indicate that R848 Mfs are not directly polarized to a type clearly identified as M1 or M2. Hadler-Olsen *et al*. [19] showed that in terms of skin and tongue carcinoma cells, the TME directs tumor progression. We thus suggest that the R848 Mfs deserve further experiments to evaluate if imiquimod modulation of TME inflammatory cells will be as beneficial in tongue cancer as it is in skin cancer.

**Fig 7:**
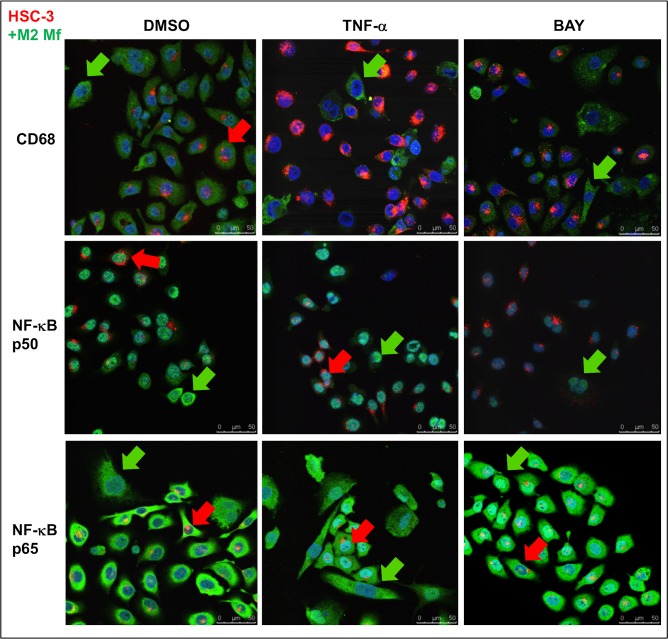
Expression of Mf-markers and NF-κB subunits in HSC-3 and M2 Mf co-culture. Vybrant CM-Dil labeled HSC-3 cells (red) and unlabeled M2 Mfs were incubated with DMSO or 10 ng/ml TNF-α for 30 min where after cells were fixed for immunofluorescence with antibodies for CD68, NF-κB p50 and p65 subunits. Some samples were pre-incubated with 10 μM BAY 11-7082 prior to TNF-α activation. AlexaFluor488-conjugated secondary antibody was used for visualization. Samples were mounted with DAPI- mountain medium to visualize nuclei (blue). Samples were photographed with a Leica Confocal microscope with 63x oil immersion objective. Red arrows indicate HSC-3 cells and green arrows Mfs. Scale bars 50 μm.

In M2 Mfs and HSC-3 cell co-cultures, CD68 positivity was seen also in HSC-3 cells and this expression was abolished by TNF-α treatment (Fig. 7, red arrow). CD163 positivity was seen only in M2 Mfs (not shown). NF-κB p50 subunit was observed in both the cytoplasm and nuclei of M2 Mfs (Fig. 7, green arrows) but in HSC-3 cells it was mostly confined to the nuclei in co-cultures (Fig. 7, red arrows). TNF-α treatment induced translocation of p50 to the nuclei of M2 Mfs (Fig. 7 green arrow) while the nuclear p50 found in HSC-3 cells was unchanged in appearance (Fig. 7, red arrow). Nuclear translocation of the NF-κB p65 subunit was observed after TNF-α treatment in HSC-3 cells (Fig. 7, red arrow) while in the M2 Mfs this translocation was almost absent (Fig. 7, green arrow). It was also lacking in HSC-3 cells in a close proximity to M2 Mfs (Fig. 7, red arrow). BAY 11-7082 abolished p50 expression in almost all cells and inhibited p65 translocation to most but not all cells (Fig. 7). It thus appears that similarly to the cultures with M2 Mfs alone, also in co-cultures the M2 Mfs are lacking in p65 translocation indicating the presence of p50 homodimers and dysregulated NF-κB activation. However, the M2 Mfs also appear to induce dysregulation of NF-κB signaling (no translocation of p65) in HSC-3 cells at least in a close proximity to the M2 Mfs. This might be one way in which TAMs add to tumor progression and also explaining why cell-cell contact appears to be important for interaction.

**Fig 8:**
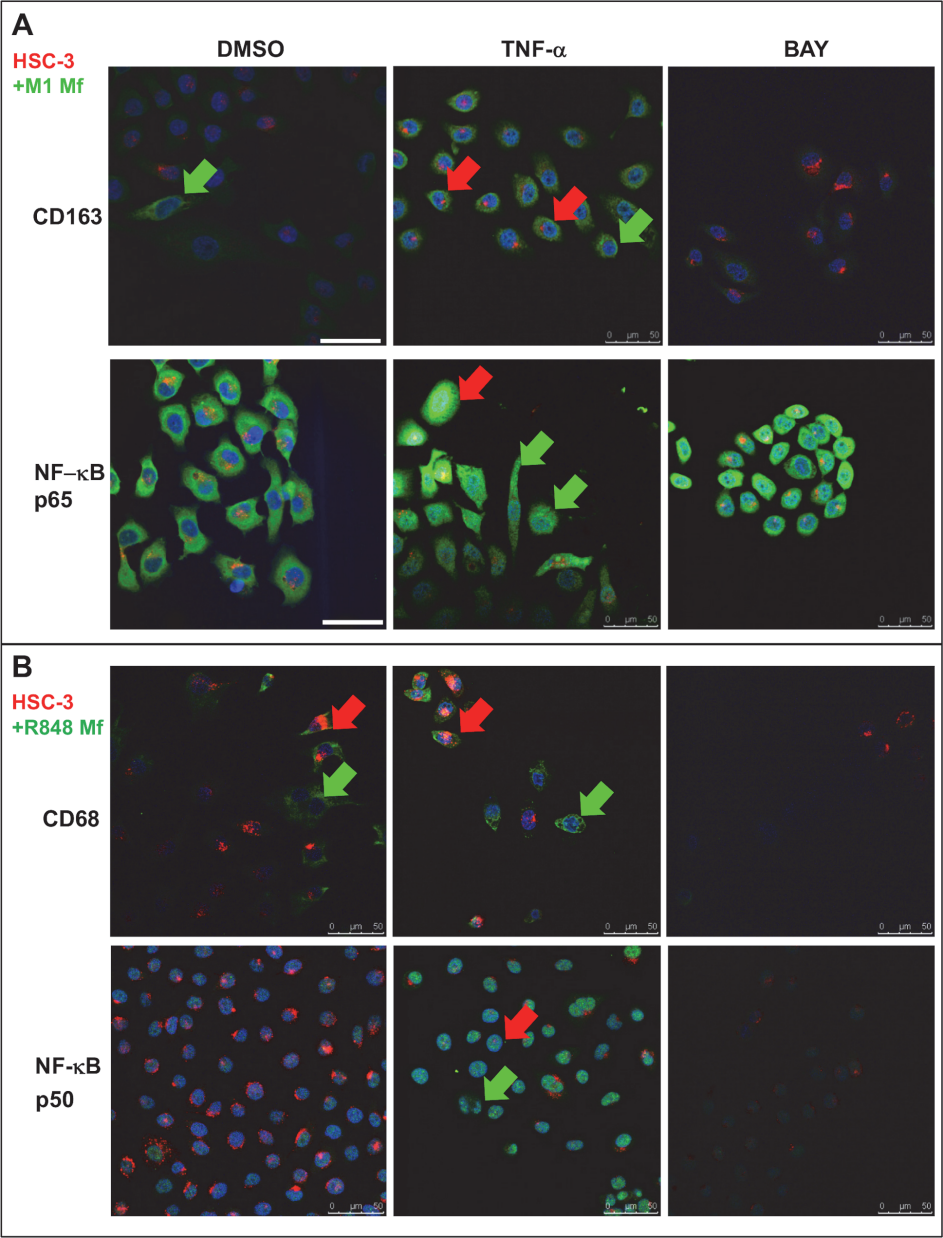
Expression of Mf-markers and NF-κB subunits in co-cultures of HSC-3 cells and M1 or R848 Mfs. Vybrant CM-Dil labeled HSC-3 cells (red) and unlabeled M1- or R848 Mfs were incubated with DMSO or 10 ng/ml TNF-α for 30 min where after cells were fixed for immunofluorescence with antibodies for CD68, CD163, NF-κB p50 and p65 subunits. Some samples were pre-incubated with 10 μM BAY 11-7082 prior to TNF-α activation. AlexaFluor488-conjugated secondary antibody was used for visualization. Samples were mounted with DAPI- mountain medium to visualize nuclei (blue). Samples were photographed with a Leica Confocal microscope with 63x oil immersion objective. CD163 and NF-κB p65 immunofluorescence from HSC-3 cell and M1 Mf co-cultures (A). CD68 and NF-κB p50 immunofluorescence from HSC-3 cell and R848 Mf co-cultures (B). Red arrows indicate HSC-3 cells and green arrows Mfs. Scale bars 50 μm.

In M1 Mfs and HSC-3 co-cultures, M1 Mfs continued to be CD68 positive (not shown), however some M1 Mfs also showed clear CD163 positivity (Fig. 8A, green arrow). Also HSC-3 cells were CD163 positive after TNF-α treatment but this was abolished after BAY treatment (Fig. 8A, red arrows). This indicates that the proximity of carcinoma cells can induce CD163 expression and polarization in M1 Mfs to TAMs. In a study by Maniecki *et al*. [60] both Mfs and cancer cells were CD163 positive in bladder cancer biopsies. In co-cultures with Mfs, the bladder cancer cells started to express CD163 indicative of either fusion or exosome RNA/protein transfer. In any case, cytokines alone were not found to induce endogenous expression of CD163 in bladder cancer cells [60]. Also breast and colorectal cancer cells are known to express CD163 which is associated with reduced patient survival compared to CD163 negative cancers [33]. Thus, CD163 expression in cancer cells is a measurement of aggressiveness. HSC-3 cells started to express CD163 after TNF-α stimulation and this may be partially NF-κB dependent since BAY 11–782 abolished the expression pattern. Therefore, CD163 could be a potentially important diagnostic tool for tongue cancer. NF-κB deposition in HSC-3 cells co-cultured with M1 Mfs appeared similar to that observed in culture without Mfs (Fig. 8A). However, M1 Mfs showed p50 deposition as seen in cultures without HSC-3 cells (S6 Fig.) while translocation of p65 was weak (Fig. 8A). This indicates that in co-cultures with M1 Mfs, HSC-3 cells retain their NF-κB activity mechanism while changing the NF-κB activity in M1 Mfs (lack of p65 translocation). Again, this indicates that HSC-3 cells in a close vicinity to M1 Mfs act to change the polarization towards a more tumor-progressive type. Co-cultures of R848 Mfs and HSC-3 cells were CD163 negative (not shown) but CD68 positive so that also HSC-3 cells were positive after TNF-α treatment (Fig. 8 B red arrows). The expression was abolished by BAY treatment. Co-culturing HSC-3 and R848 Mfs resulted in reduced p50 appearance in both cell types (Fig. 8B) while TNF-α treatment increased expression and BAY 11–782 abolished it. NF-κB p65 was readily detected in the cytoplasm of HSC-3 and R848 Mfs in co-culture (S6 Fig.). Nuclear translocation was observed in HSC-3 cells after TNF-α treatment but with less nuclear deposition in R848 Mfs. BAY 11-7082 almost completely blocked translocation (S6 Fig.).

These findings indicate that co-culturing R848 Mfs with HSC-3 cells changes the expression and deposition of p50 subunit while p65 deposition and nuclear translocation appears rather normal.

**Fig 9:**
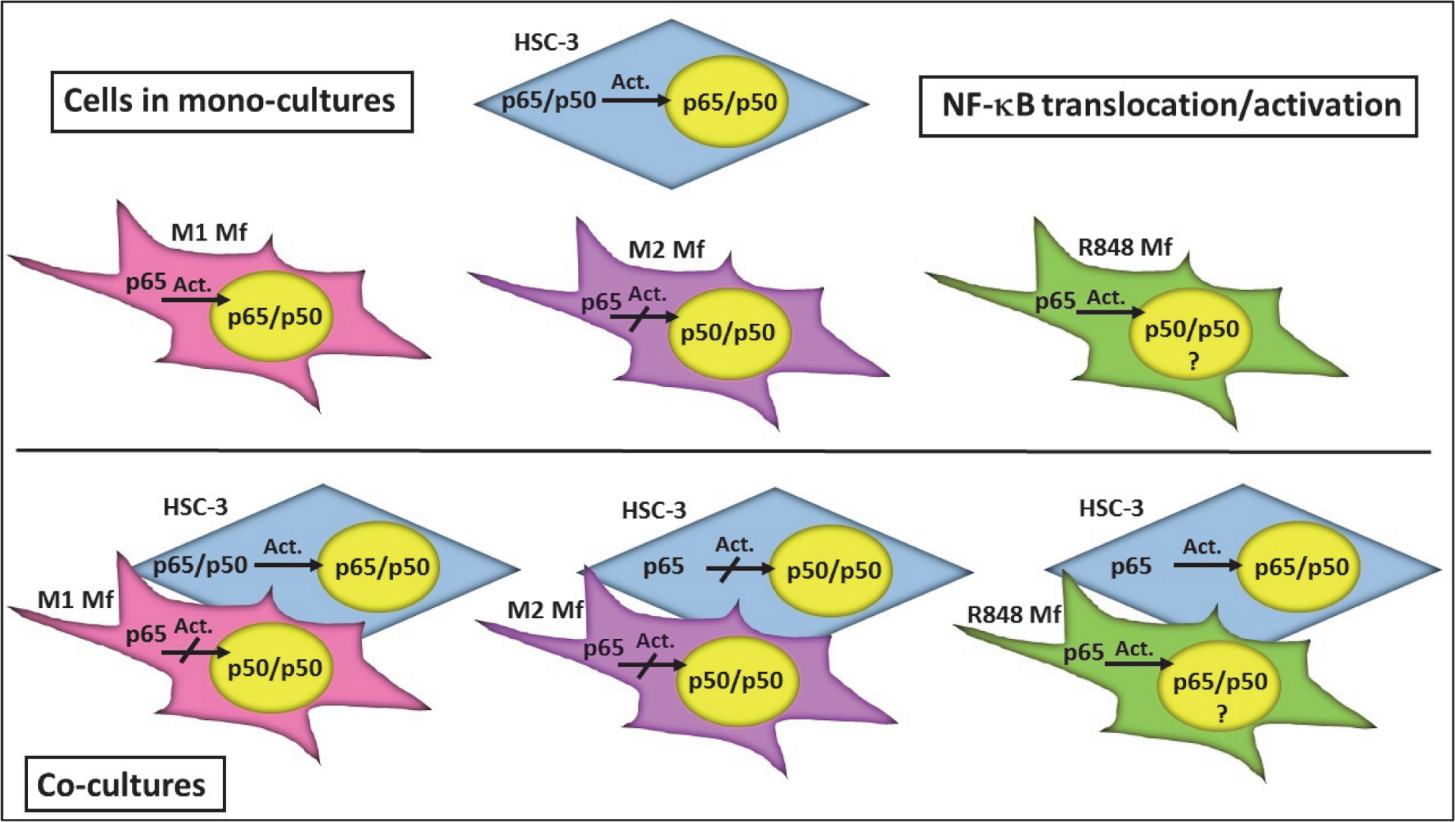
Summary of NF-κB translocation/activation in HSC-3 cell and Mfs co-cultures. In monocultures, a typical activation of the NF-κB dimer (p65/p50 translocation to nucleus) is seen in HSC-3 cells and M1 Mfs while M2 Mfs present with a p50/p50 homodimer pattern also present in R848 Mfs. In co-cultures, HSC-3 cells induced a shift in M1 Mfs NF-κB activation towards a p50/p50 profile. In HSC-3 and M2 Mfs co-cultures on the other hand, NF-κB activation in the HSC-3 cells was shifted if the HSC-3 cells were in a close proximity to the M2 Mfs. The NF-κB activation profile of R848 Mfs in co-cultures was more cryptic but translocation of p50/p65 could be observed. The p65/p50 heterodimer activates NF-κB inducible genes while the p50/p50 homodimer is associated with repressed gene transcription or induction of tumor-progressive genes.

The results from the NF-κB activation experiment are summarized in Fig. 9.

### The role of TME for macrophage migration *in vivo*

**Fig 10:**
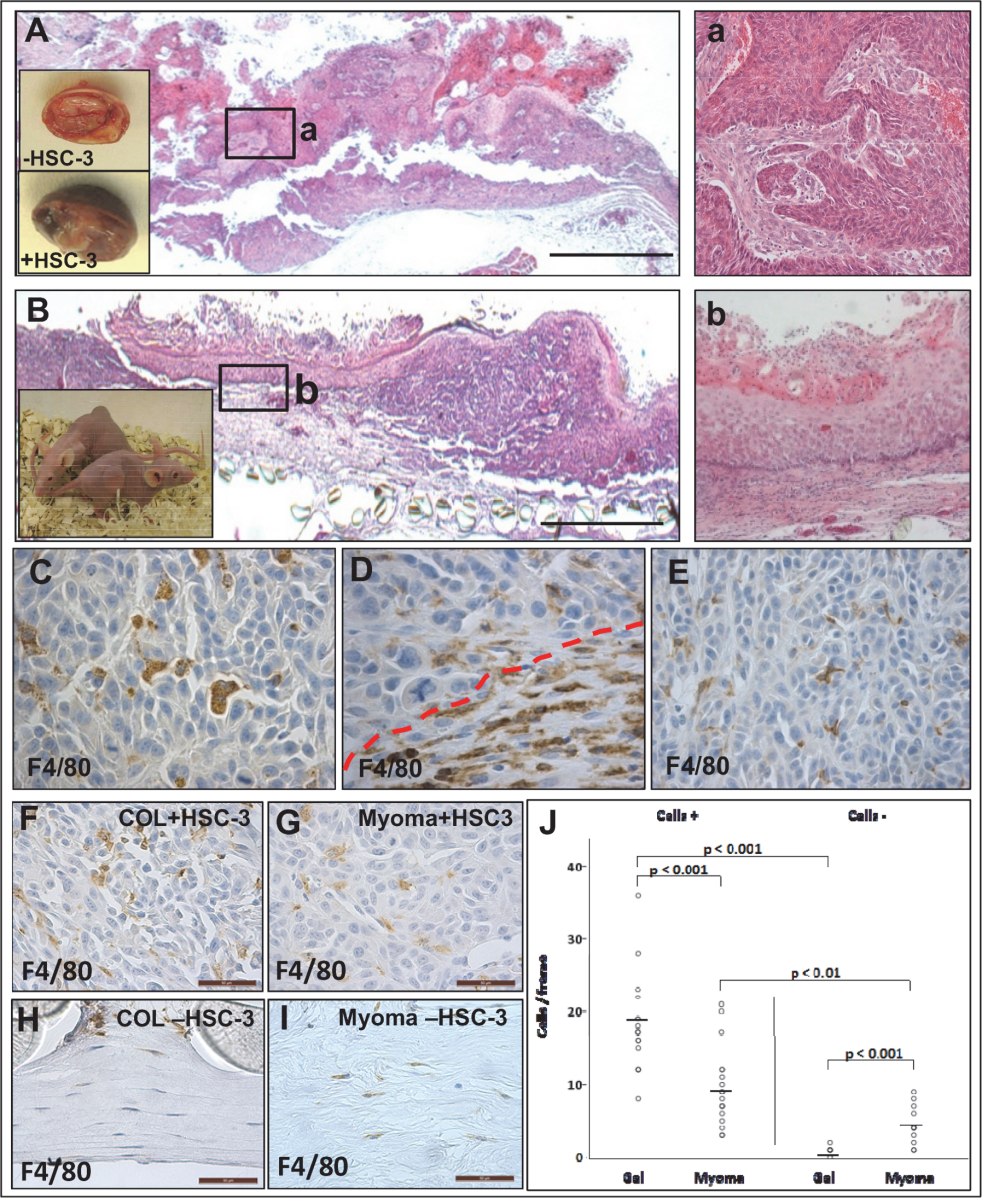
Mfs migrate to transplanted myoma tissue in nude mice. Myoma tissue or collagen gels with fibroblasts and with or without HSC-3 cells were transplanted into nude mice (small insert figure in B, n = 5). Appearance of myoma after transplantation with and without HSC-3 cells as small inserts in A. After removal, tissues were processed for routine H&E staining (A,B, a,b) and immunohistochemistry with mouse macrophage marker F4/80 (C-E). Overview of myoma-HSC-3 cells tissue (A) and invasive area in (a). Overview of collagen gel-HSC-3 cells (B) and invasive area in (b). Scale bars 100 μm. Mfs migrated into the myoma-HSC-3 tumors (C,E) as well as to the myoma-mouse tissue interface (D, interface marked with red dotted line). Analysis of Mfs in tissue sections from the gel or myoma—mouse host tissue interface (F-J). All images were photographed using a Leica microscope-camera system. Scale bars 50 μm. The Mfs counted from the myoma/gel stroma using ImageJ software (J).

To study the role of TME *in vivo*, myomas and collagen gels containing gingival fibroblasts with or without HSC-3 cells were transplanted into the back of nude mice (depicted in the insert in Fig. 10B). HSC-3 cells on myoma tissue generated a stromal reaction in the mice and invasion could be observed (Fig. 10A, magnification in a). HSC-3 cells transplanted on collagen gels were also found to invade to some extent; however the stromal reaction was lacking (Fig. 10B, magnification in b). Transplanted myoma tissue reactions with HSC-3 cells (Fig. 10, insert in A) were more necrotic and cancer-like in appearance than benign myoma tissue implants without HSC-3 cells (Fig. 10, insert in A).

Mouse F4/80 positive-Mfs had migrated to the transplanted human myoma-HSC-3 tumor tissue (Fig. 10C,E). Mfs within the tumor tissue were polyglonal in shape and some were granular (Fig. 10C,E). Mfs at the tumor-mouse tissue interface were abundant and more elongated in shape (Fig. 10D, interface marked with red line). Mouse Mfs were found to migrate also to the transplanted collagen gel with or without HSC-3 cells (Fig. 10 F,H). In addition, Mfs also migrated to the cell-free transplanted myoma tissue in a similar fashion as to the myomas with HSC-3 cells (Fig. 10 G,I). The Mfs were studied in more detail by counting cells both in the tumor and at the tumor-mouse tissue interface. No differences in macrophage number was detected in the dense macrophage area at the gel/myoma—mouse tissue interface (not shown). However, the dense area contained significantly more Mfs when the gel/myoma contained HSC-3 cells compared to gel/myoma tissue alone (not shown). When the gel/myoma tissue was analysed for Mfs (by counting the F4/80-positive cells), it was again observed that the gel/myoma containing HSC-3 cells also contained significantly more Mfs than cell-free gel/myoma (Fig. 10 J, p<0.001 for both). Interestingly, while the collagen gel containing HSC-3 cells also contained more Mfs than the myoma tissue (Fig. 10 J, p<0.001), the cell-free myoma tissue contained significantly more Mfs than the cell-free collagen gel (Fig. 10 J, p<0.001). These results show that in an *in vivo* setting, the TME itself act as a chemoattractant for Mfs but with tumor cells present, the effect is even more profound. In addition, while the Mfs more easily penetrate the collagen gel than myoma tissue when HSC-3 cells serve as chemoattractants, the cell-free myoma tissue itself contains pro-migratory signals for Mfs that are lacking in the artificial collagen gel. These observations reinforce our previous observations that myoma tissue mimics the TME well.

### TAMs are localized to evading carcinoma cells in tongue carcinoma tissue

**Fig 11:**
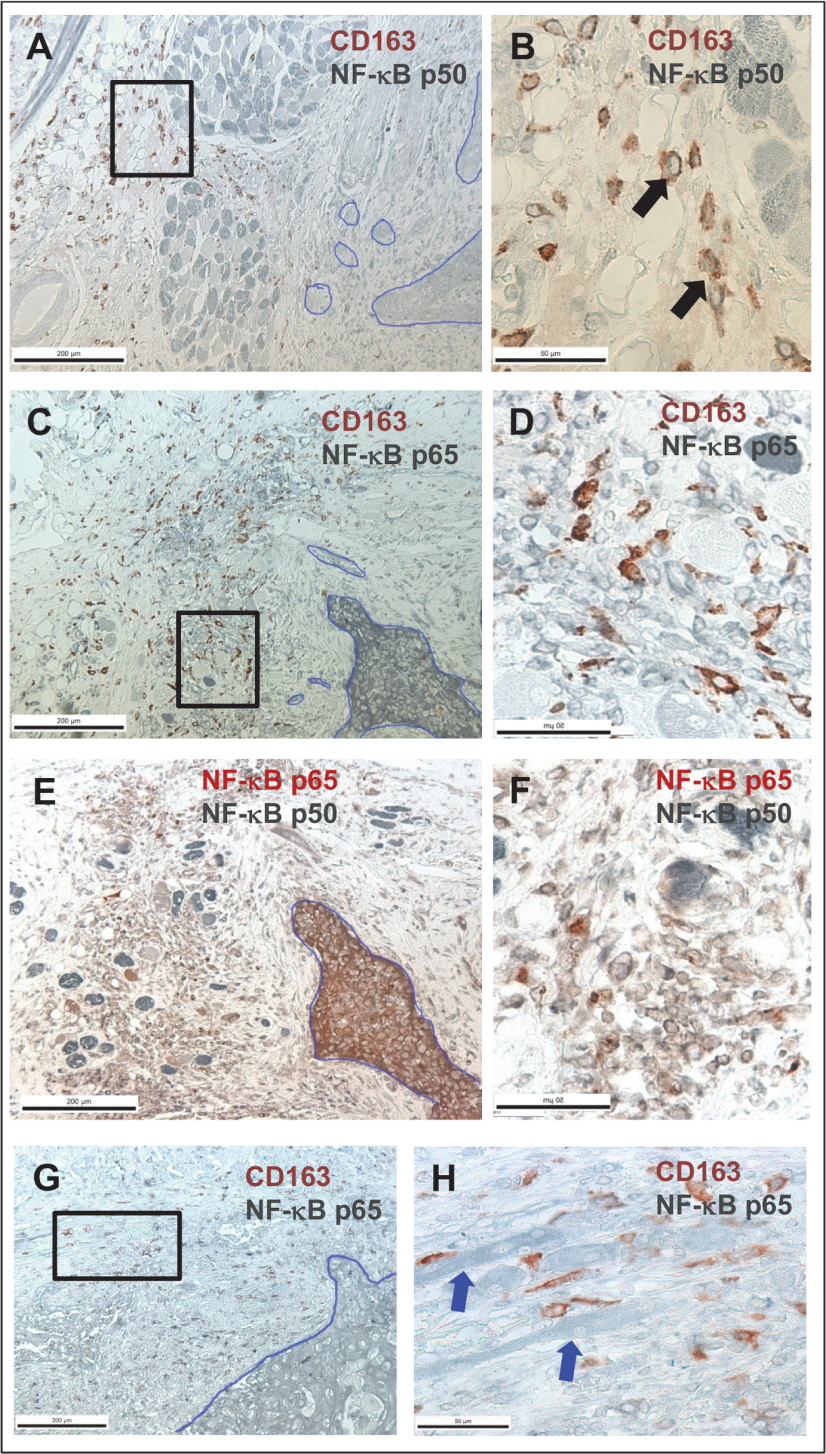
TAMs localize to evading carcinoma cells *in vivo*. Human tongue carcinoma tissue sections were subjected to double-staining immunohistochemistry with antibodies for CD163, NF-κB p50 and NF-κB p65. CD163 (red) and NF-kB p50 (grey) staining in A, B. Black arrows show cells with both red and grey color, indicating co-localization of the antibodies. CD163 (red) and NF-κB p65 (grey) staining in C,D, G,H. Blue arrows in H show tongue carcinoma cells. NF-kB p50 (grey) and NF-kB p65 (red) staining in E,F. Black boxes in A, C and G indicates site of magnification in B, D and H respectively. Scale bars in A,C,E and G 200 μm and in B, D, F, and H 50 μm.

To define the role of TAMs more precisely *in vivo*, we stained human tongue carcinoma tissue sections obtained from cancer patients at the Oulu University Hospital, Finland. Tissue sections were double-stained with antibodies for TAMs (CD163) and NF-κB subunits p50 and p65. CD163-positive cells were found in a close vicinity to invasive carcinoma cell islands (Fig. 11, depicted with a blue line), leading the way into the muscular layer (Fig. 11 A-D). CD163-positive cells were also abundant around evading carcinoma cells (Fig. 11 C,D,G,H). In some areas, CD163-positive cells appeared to be in direct contact with evading carcinoma cells (Fig. 11 G,H, blue arrows indicating carcinoma cells). Interestingly, while NF-κB p65 was expressed in carcinoma tissue (Fig. 11 C-F), NF-κB p50 was lacking or much less abundant (Fig. 11 A,B,E,F). However, not all carcinoma tissue showed strong staining for NF-κB p65 (Fig. 11 G,H). On the other hand, while most CD163-positive cells were negative for NF-κB p65 staining (Fig. 11 D,H), expression for NF-κB p50 was detected (Fig. 11 B,black arrows). Double-staining for both p50 and p65 showed that while the carcinoma tissue was mostly expressing p65, the stromal cells were expressing either subunit or both subunits (Fig. 11 E,F). Some areas of the carcinoma lesions did not show any CD163 positive cells or NF-κB expression (not shown). Tissue sections treated with non-immune serum showed no staining (S7 Fig.). These results indicate that while the carcinoma tissue itself expresses NF-κB p65, there is less expression of NF-κB p50 while the situation is reversed in TAMs, further evidencing the existence of p50 homodimer formation in TAMs. A high number of TAMs are associated with metastasis and correlate with low survival in OSCC [18,61]. In addition, several studies show that primary HNSCC tumors with high number of TAMs are associated with lymph node metastasis, extracellular capsular spread and advanced stage [36,62]. Our findings corroborate previous studies and further strengthens the paradigm of using CD163 as a diagnostic tool for OSCC.

## Conclusions

We here show for the first time that HSC-3 cells fuse with Mfs and especially the interaction between HSC-3 cells and M2 Mfs induced migration and also invasion in the 3D myoma model. Co-culturing altered the phenotype of both HSC-3 cells and Mfs as well as completely altered the cytokine secretion profile. Especially the expression of EGF, M-CSF and TGF-, known to act pro-tumorigenic in OSCC, were induced in co-cultures with HSC-3 cells and M2 Mfs. In addition, M2 Mfs were found to contain an excess of NF-κB p50 homodimers which can be critical for oral tumor progression. *In vivo*, HSC-3 cells and to a lesser extent also the TME in myoma tissue, attracted Mfs and in addition, tongue cancer patient tissue sections are redundant in M2 Mfs. These results show that TAMs are actively involved in tongue cancer progression and should be considered as therapeutic targets in tongue cancer treatment.

## Supporting Information

S1 Table Short Tandem Repeat (STR) profiling of commercial HSC-3 cells purchased from the Japanese Collection of Research Bioresources Cell Bank, Japan. HSC-3 catalogue number: JCRB0623. The STR-profiling was done by Identicell, Aarhus, Denmark.
(PDF)

S2 Table Short Tandem Repeat (STR) profiling of commercial THP-1 cells purchased from ATCC (cat.no. ATCC TIB-202). The STR-profiling was done by Identicell, Aarhus, Denmark.
(PDF)

S1 Dataset Serum-free media from HSC-3 cells, Mfs and co-cultures were collected at day 2 and analyzed for cytokines with RayBio Human Cytokine Antibody Array G5. The table shows the raw data of signal intensity from the antibody array.
(XLS)

S1 Fig (A) Relative Mf cell density in co-cultures. Vybrant CM-Dil-labeled HSC-3 cells (red) and Vybrant DiO-labeled Mfs (green) were co-cultured for up to 11 days in normal growth medium and photographed with an Evos FL Cell Imaging System microscope. Cell density was analyzed optically using Leica QWin3 Software. (B-E) Vybrant CM-Dil labeled HSC-3 (red) and Vybrant DiO-labeled macrophages (green) were co-cultured in Oris Pro cell migration 96-plates. Inserts were then removed and cells allowed to migrate into the empty space. Cells were photographed with an EVOS FL Cell Imaging System microscope. HSC-3 and R848 Mfs migration at 24 hours (B) and magnification from the migratory front in B (white box) shown in C. HSC-3 and M1 Mfs migration at 48 hours in (D) and magnification from the migratory front in D (white box) shown in E. White arrows indicate merged cells. Scale bars in B,D: 1000 μm and in C,E: 200 μm. n = 6.
(TIF)

S2 Fig Mfs were seeded to the upper chamber of Transwell-inserts and HSC-3 cells were seeded to the lower chambers in SF-medium (A). Cells were allowed to migrate for 24 hours and then cells were stained with Crystal violet, photographed and analysed with Leica Qwin3 software. Results are presented as mean area of cells in inserts (n = 4). HSC-3 cells and R848 Mfs were co-cultured on top of human myoma tissue (B,C) or HSC-3 cells were cultured on top of myoma tissue treated with NF-κB inhibitor BAY 11-7082 (10 μM) or R848 solution (50 nM) which was also added to the incubation medium (B,C). HSC-3 cells were cultured on top of myoma tissues using Mf-CMs as incubation media (D). Incubation was continued for 10 days where after tissues were fixed and processed for immunohistochemistry. Pan-cytokeratin stained sections were photographed and invasion depths (B,D) and invasion indexes (C) were analysed with the Leica Qwin3 software. All myoma experiments were done in triplicate.
(TIF)

S3 Fig Conditioned medium was collected at day 4 and 8 from myomas and medium containing 0.5 (left) or 15 μg (right) protein were subjected to gelatin zymography. The figure to the left shows the uncropped zymogram from day 4 myoma medium with 0.5 μg loaded protein which is presented slightly cropped in Fig. 5E to be more representative and more easily interpreted. The figure to the right shows the uncropped zymogram from day 4 myoma medium with 14 μg loaded protein to show the gelatinases in the HSC-3 sample which were not visible when loading 0.5 μg protein.
(TIF)

S4 Fig Vybrant CM-Dil labeled HSC-3 cells (red) and unlabeled M1-, M2 and R848 Mfs were incubated with DMSO where after cells were fixed for immunofluorescence with antibodies for CD68, CD163 and pancytokeratin. AlexaFluor488-conjugated secondary antibody was used for visualization. Samples were mounted with DAPI- mounting medium to visualize nuclei (blue). Samples were photographed with a Leica Confocal microscope with 63x oil immersion objective. CD68 marker staining in DMSO-treated M1 Mfs (A), M2 Mfs (B) and R848 Mfs (C). CD163 marker staining in DMSO-treated M2 Mfs treated (D) and R848 Mfs (E). DMSO-treated HSC-3 cells (red) stained with pancytokeratin (green) in F. Scale bars 50 μm.
(TIF)

S5 Fig Unlabeled M1 and R848 Mfs were incubated with DMSO or 10 ng/ml TNF-α for 30 min where after cells were fixed for immunofluorescence with polyclonal NF-κB p50 (A) or p65 antibody (B). Some samples were pre-incubated with 10 μM BAY 11-7082 prior to TNF-α activation. AlexaFluor488-conjugated secondary antibody was used for visualization. Samples were mounted with DAPI- mountain medium to visualize nuclei (blue). Samples were photographed with a Leica Confocal microscope with 63x oil immersion objective. Scale bars 50 μm.
(TIF)

S6 Fig Vybrant CM-Dil labeled HSC-3 cells (red) and unlabeled M1-and R848 Mfs were incubated with DMSO or 10 ng/ml TNF-α for 30 min where after cells were fixed for immunofluorescence with antibodies for NF-κB p50 and p65 subunits. Some samples were pre-incubated with 10 μM BAY 11-7082 prior to TNF-α activation. AlexaFluor488-conjugated secondary antibody was used for visualization. Samples were mounted with DAPI- mountain medium to visualize nuclei (blue). Samples were photographed with a Leica Confocal microscope with 63x oil immersion objective. Red arrows indicate HSC-3 cells (red) and green arrows macrophages (unlabeled). Scale bars 50 μm.
(TIF)

S7 Fig Control immunohistochemical staining of human tongue carcinoma tissue sections. Non-specific mouse immunoglobulins (A), rabbit immunoglobulins (B) and both mouse and rabbit immunoglobulins (C). Scale bars 200 μm.
(TIF)
