# Supplementary material for: Macrophages Modulate Migration and Invasion of Human Tongue Squamous Cell Carcinoma
Source: PLoS One. 2015 Mar 26;10(3):e0120895. doi: 10.1371/journal.pone.0120895 (PMC4374792; doi:10.1371/journal.pone.0120895)
Supplement: S1 Table — HSC-3 catalogue number: JCRB0623. The STR-profiling was done by Identicell, Aarhus, Denmark. (PDF) [file pone.0120895.s001.pdf]

Cell Line Authentication
IdentiCell STR allele report

Department of Molecular Medicine

Aarhus University Hospital

Brendstrupgaardsvej 100, 8200 Aarhus N

Denmark

Phone: +45 784 55305 email: contact@identicell.dk

www.identicell.eu

**IC-Id:**	IC-057
**Date:**	2014-06-04 08:40:07.0
**Sample:**	HSC-3

Marker	Allele(s)	
AMEL	X	Y
CSF1PO	11	
D13S317	12	
D16S539	9	
D21S11	30	31.2
D5S818	11	13
D7S820	13	
TH01	6	9.3
TPOX	8	
vWA	14	17

Comments: 100% match with HSC-3
