# Supplementary figures and images for: Macrophages Modulate Migration and Invasion of Human Tongue Squamous Cell Carcinoma
Source: PLoS One. 2015 Mar 26;10(3):e0120895. doi: 10.1371/journal.pone.0120895 (PMC4374792; doi:10.1371/journal.pone.0120895)

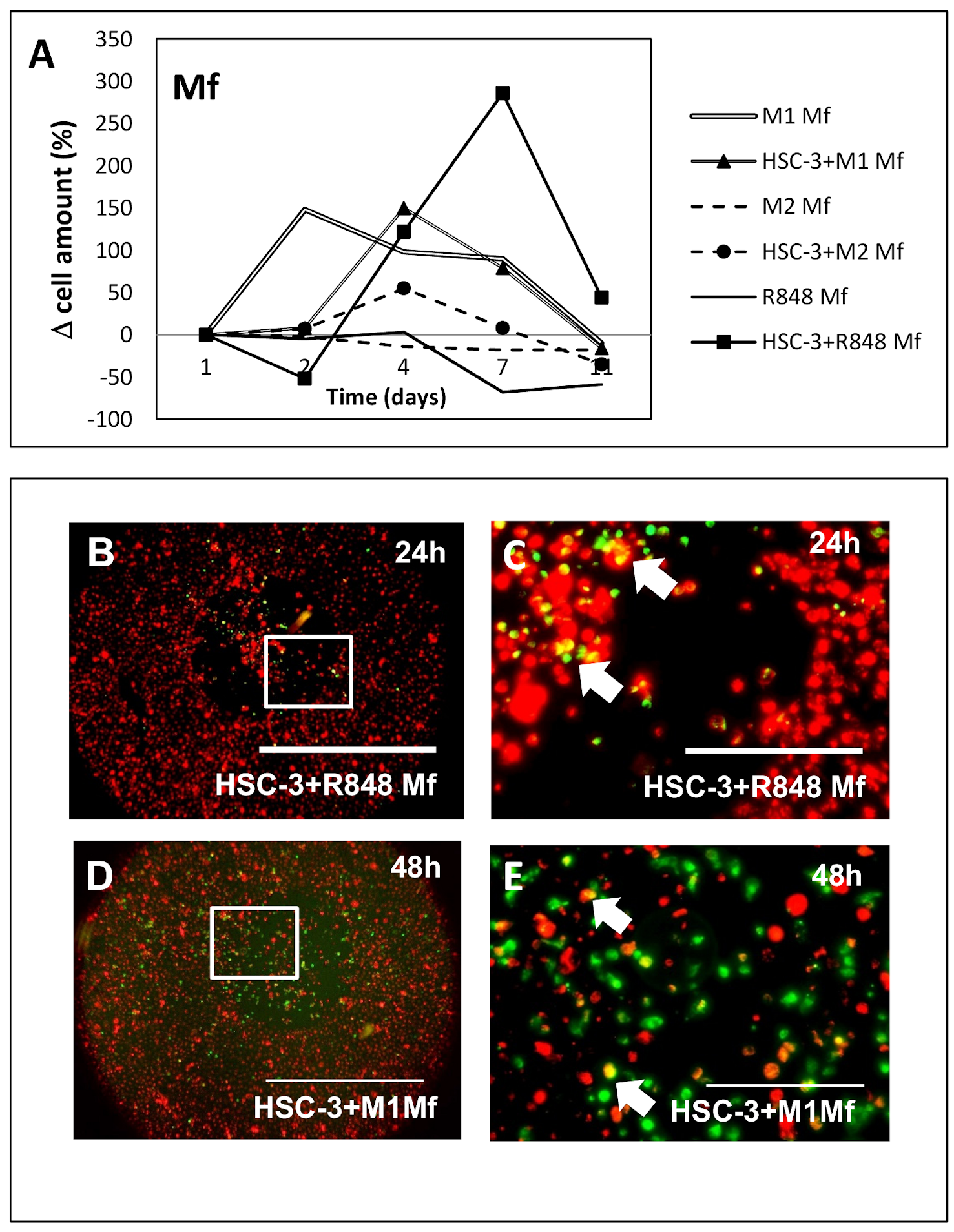

Supplement: S1 Fig — Vybrant CM-Dil-labeled HSC-3 cells (red) and Vybrant DiO-labeled Mfs (green) were co-cultured for up to 11 days in normal growth medium and photographed with an Evos FL Cell Imaging System microscope. Cell density was analyzed optically using Leica QWin3 Software. (B-E) Vybrant CM-Dil labeled HSC-3 (red) and Vybrant DiO-labeled macrophages (green) were co-cultured in Oris Pro cell migration 96-plates. Inserts were then removed and cells allowed to migrate into the empty space. Cells were photographed with an EVOS FL Cell Imaging System microscope. HSC-3 and R848 Mfs migration at 24 hours (B) and magnification from the migratory front in B (white box) shown in C. HSC-3 and M1 Mfs migration at 48 hours in (D) and magnification from the migratory front in D (white box) shown in E. White arrows indicate merged cells. Scale bars in B,D: 1000 μm and in C,E: 200 μm. n = 6. (TIF) [file pone.0120895.s004.tif]

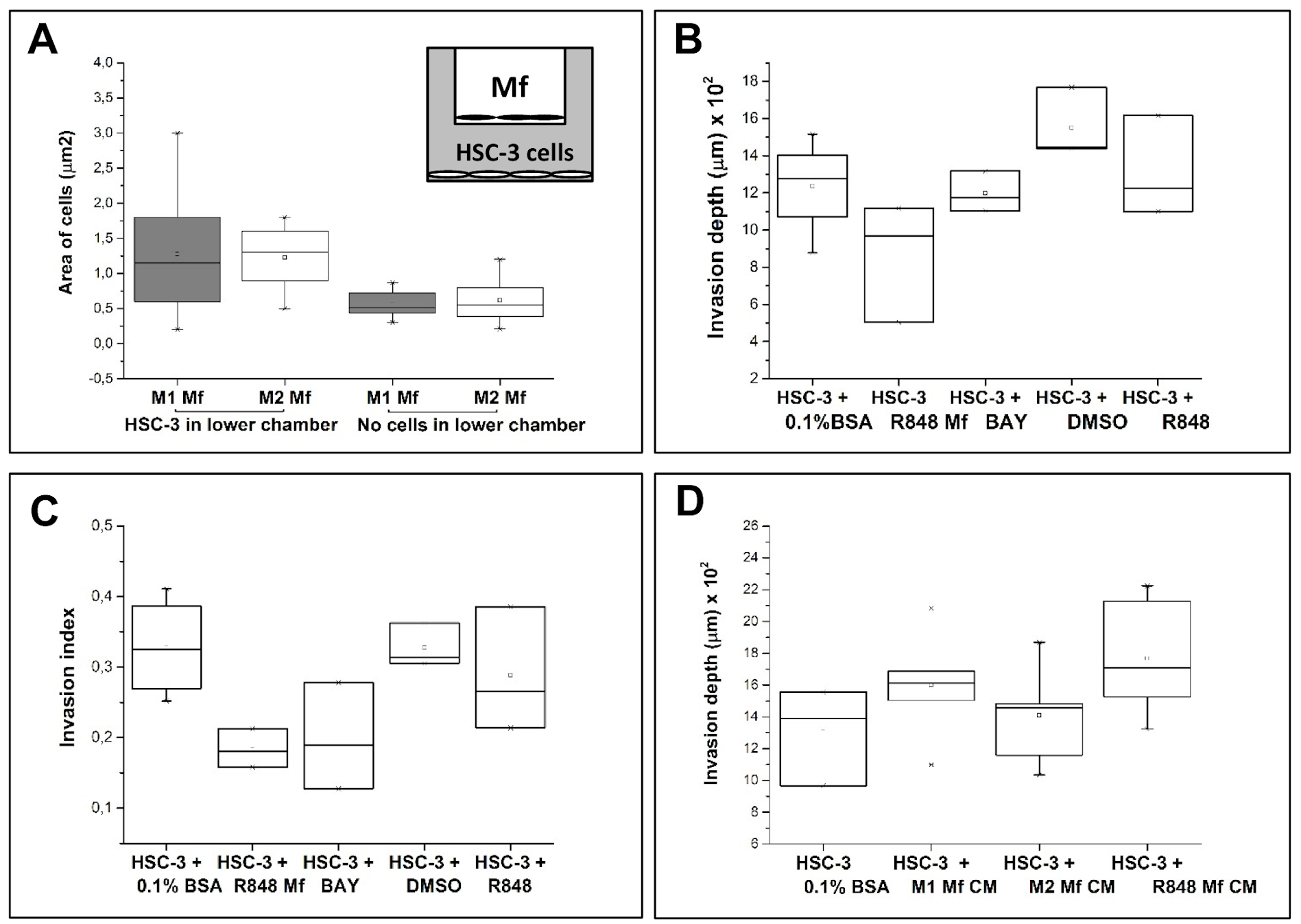

Supplement: S2 Fig — Cells were allowed to migrate for 24 hours and then cells were stained with Crystal violet, photographed and analysed with Leica Qwin3 software. Results are presented as mean area of cells in inserts (n = 4). HSC-3 cells and R848 Mfs were co-cultured on top of human myoma tissue (B,C) or HSC-3 cells were cultured on top of myoma tissue treated with NF-κB inhibitor BAY 11-7082 (10 μM) or R848 solution (50 nM) which was also added to the incubation medium (B,C). HSC-3 cells were cultured on top of myoma tissues using Mf-CMs as incubation media (D). Incubation was continued for 10 days where after tissues were fixed and processed for immunohistochemistry. Pan-cytokeratin stained sections were photographed and invasion depths (B,D) and invasion indexes (C) were analysed with the Leica Qwin3 software. All myoma experiments were done in triplicate. (TIF) [file pone.0120895.s005.tif]

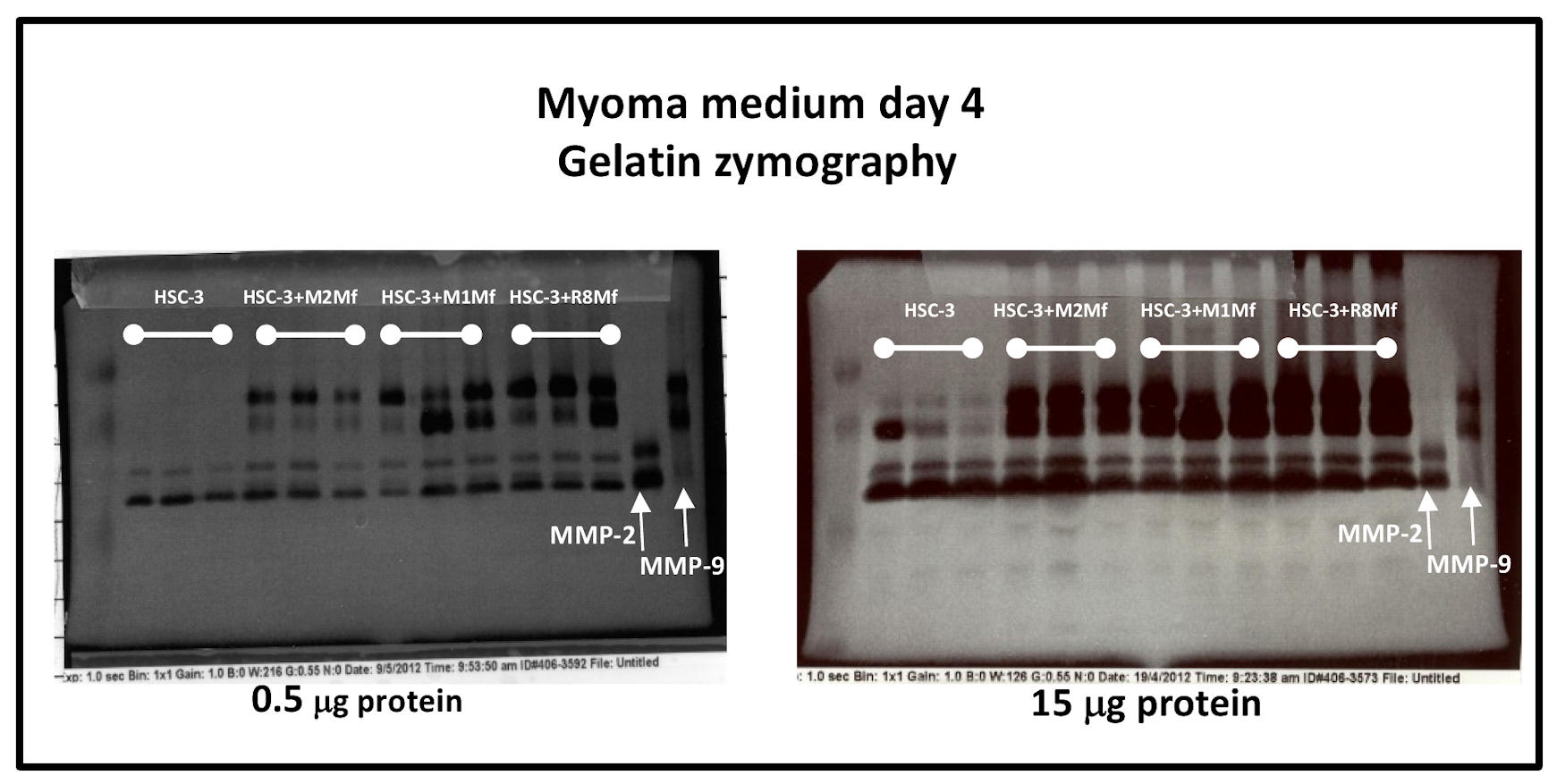

Supplement: S3 Fig — The figure to the left shows the uncropped zymogram from day 4 myoma medium with 0.5 μg loaded protein which is presented slightly cropped in Fig. 5E to be more representative and more easily interpreted. The figure to the right shows the uncropped zymogram from day 4 myoma medium with 14 μg loaded protein to show the gelatinases in the HSC-3 sample which were not visible when loading 0.5 μg protein. (TIF) [file pone.0120895.s006.tif]

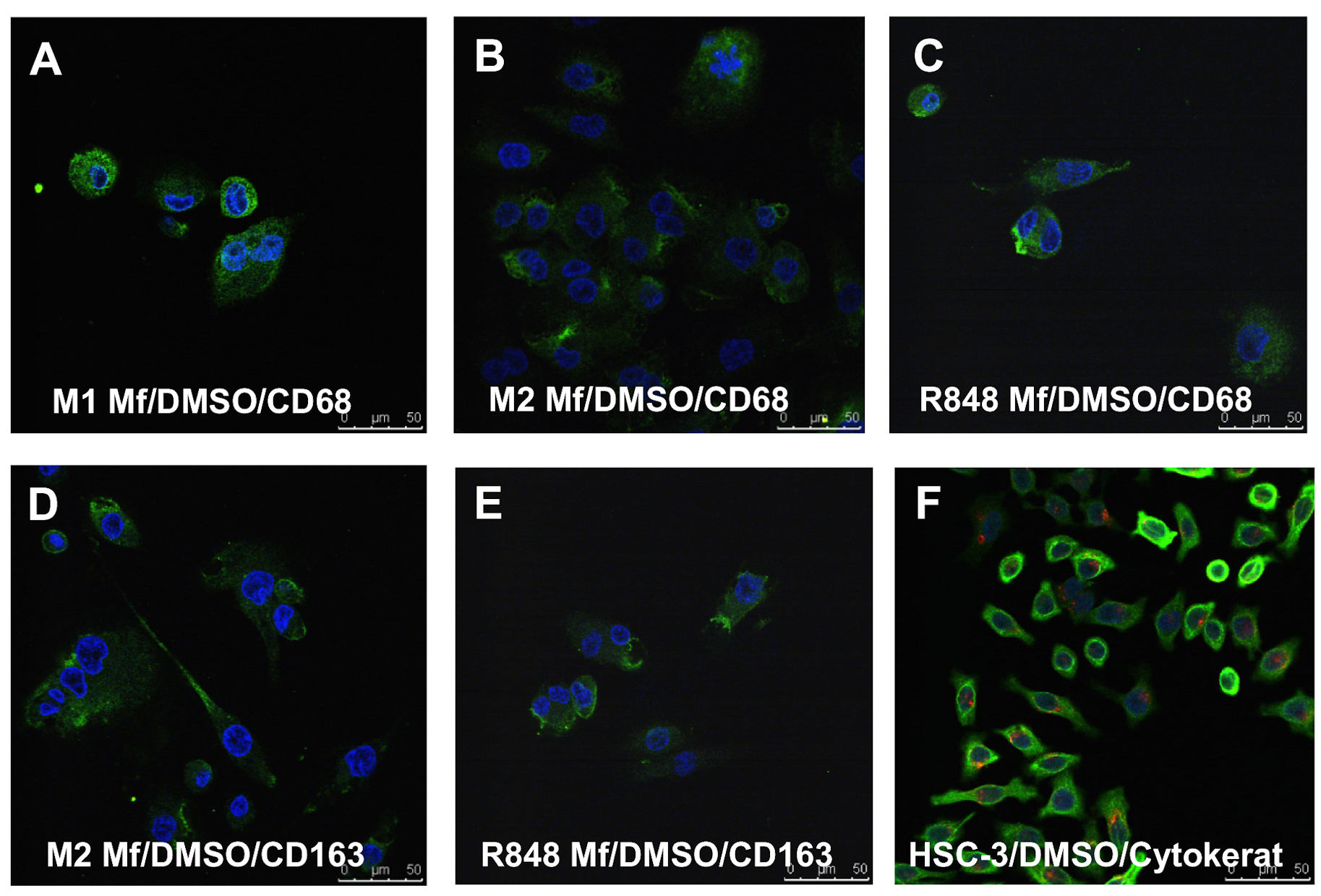

Supplement: S4 Fig — AlexaFluor488-conjugated secondary antibody was used for visualization. Samples were mounted with DAPI- mounting medium to visualize nuclei (blue). Samples were photographed with a Leica Confocal microscope with 63x oil immersion objective. CD68 marker staining in DMSO-treated M1 Mfs (A), M2 Mfs (B) and R848 Mfs (C). CD163 marker staining in DMSO-treated M2 Mfs treated (D) and R848 Mfs (E). DMSO-treated HSC-3 cells (red) stained with pancytokeratin (green) in F. Scale bars 50 μm. (TIF) [file pone.0120895.s007.tif]

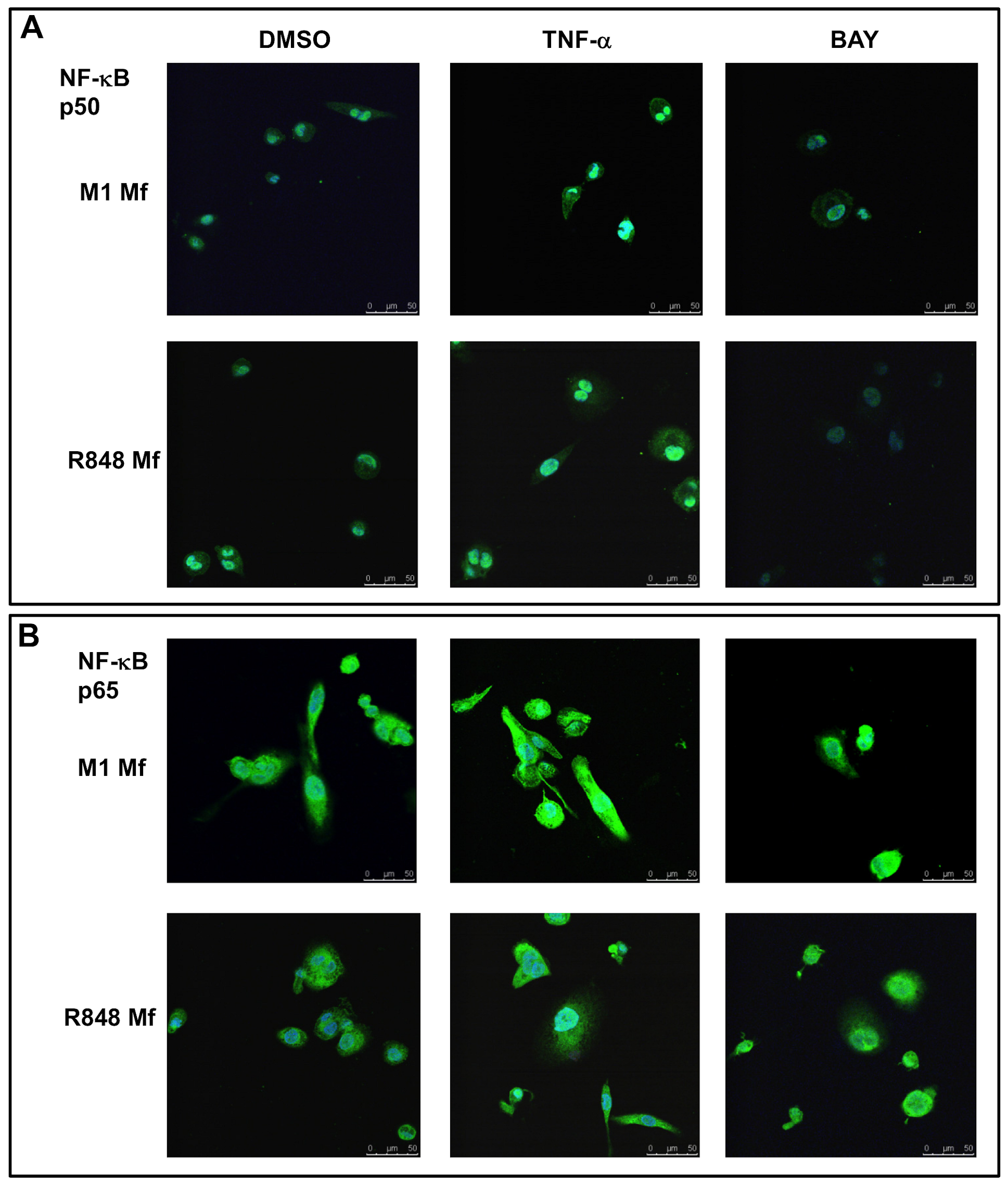

Supplement: S5 Fig — Some samples were pre-incubated with 10 μM BAY 11-7082 prior to TNF-α activation. AlexaFluor488-conjugated secondary antibody was used for visualization. Samples were mounted with DAPI- mountain medium to visualize nuclei (blue). Samples were photographed with a Leica Confocal microscope with 63x oil immersion objective. Scale bars 50 μm. (TIF) [file pone.0120895.s008.tif]

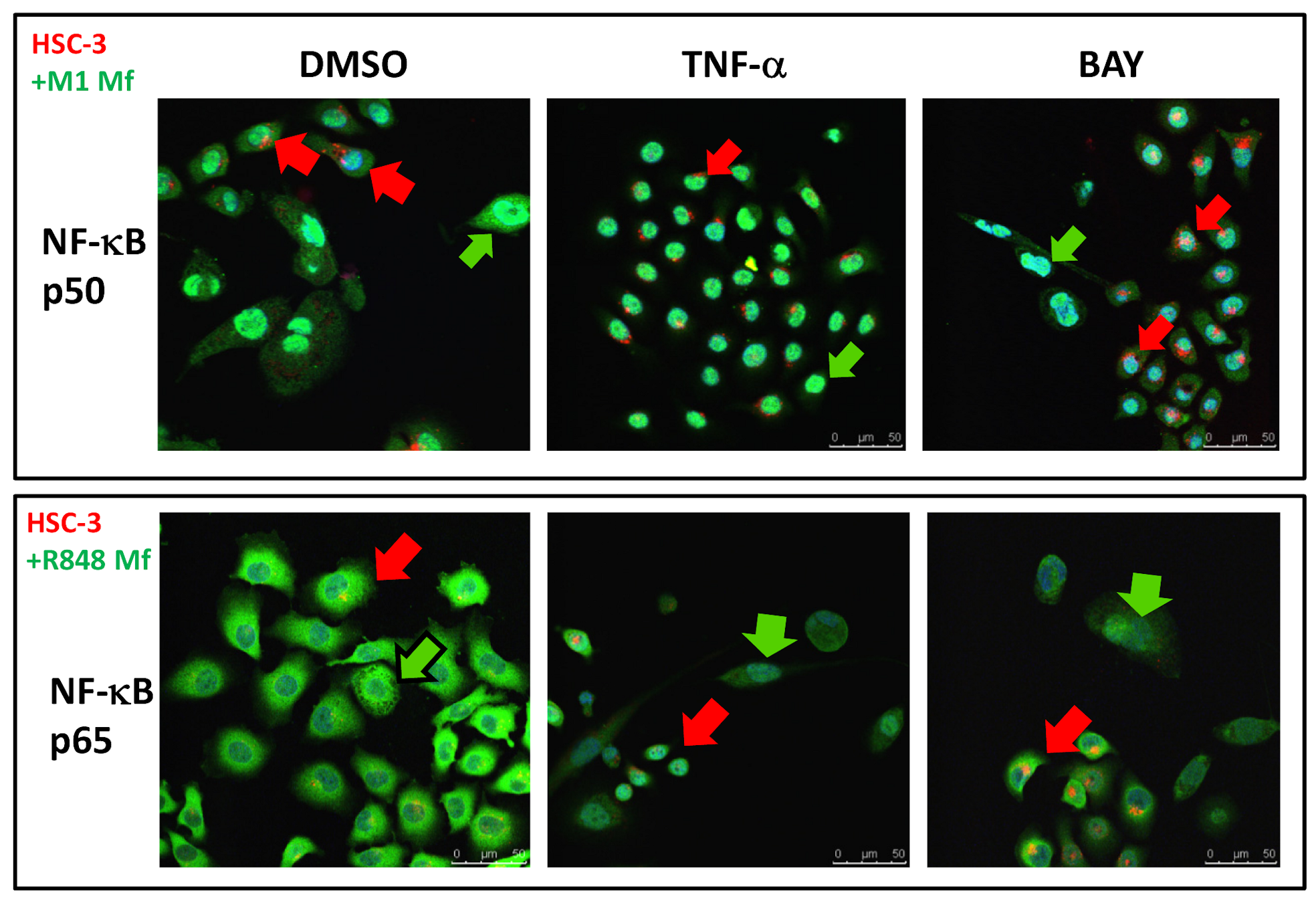

Supplement: S6 Fig — Some samples were pre-incubated with 10 μM BAY 11-7082 prior to TNF-α activation. AlexaFluor488-conjugated secondary antibody was used for visualization. Samples were mounted with DAPI- mountain medium to visualize nuclei (blue). Samples were photographed with a Leica Confocal microscope with 63x oil immersion objective. Red arrows indicate HSC-3 cells (red) and green arrows macrophages (unlabeled). Scale bars 50 μm. (TIF) [file pone.0120895.s009.tif]

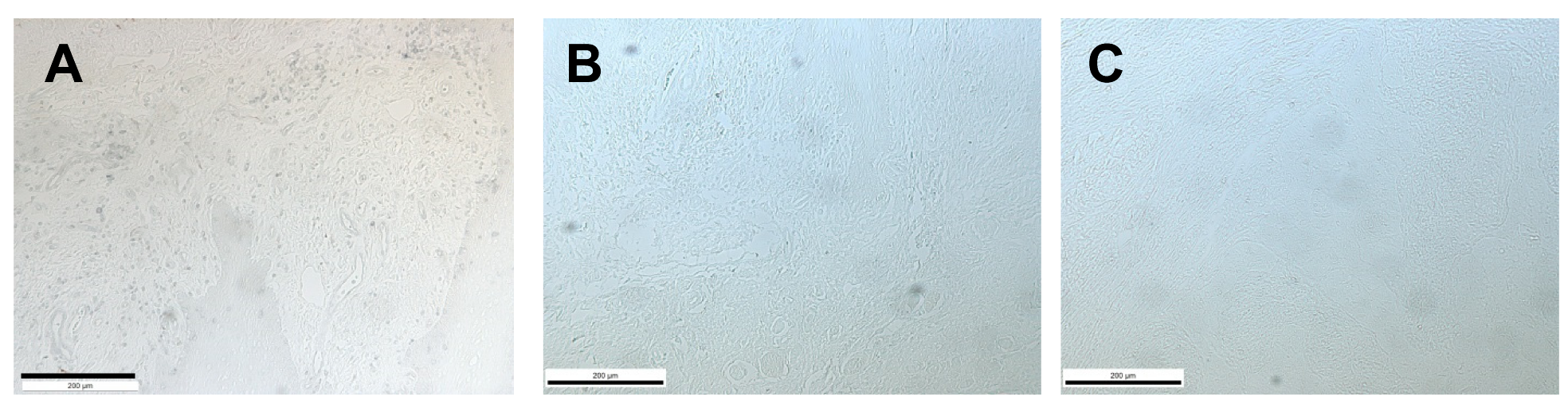

Supplement: S7 Fig — Non-specific mouse immunoglobulins (A), rabbit immunoglobulins (B) and both mouse and rabbit immunoglobulins (C). Scale bars 200 μm. (TIF) [file pone.0120895.s010.tif]
